# The dePARylase NUDT16 promotes radiation resistance of cancer cells by blocking SETD3 for degradation *via* reversing its ADP-ribosylation

**DOI:** 10.1016/j.jbc.2024.105671

**Published:** 2024-01-23

**Authors:** Weijun Wu, Wenjing Wu, Yingshi Zhou, Qiao Yang, Shuting Zhuang, Caixia Zhong, Wenjia Li, Aixin Li, Wanzhen Zhao, Xiaomin Yin, Xuyu Zu, Carmen Chak-Lui Wong, Dong Yin, Kaishun Hu, Manbo Cai

**Affiliations:** 1 Guangdong Provincial Key Laboratory of Malignant Tumor Epigenetics and Gene Regulation, Guangdong-Hong Kong Joint Laboratory for RNA Medicine, Medical Research Center, Sun Yat-Sen Memorial Hospital, Sun Yat-Sen University, Guangzhou, China; 2 Department of Oncology Radiotherapy, The First Affiliated Hospital, Hengyang Medical School, University of South China, Hengyang, Hunan, China; 3 Department of Breast Oncology, Sun Yat-Sen Memorial Hospital, Sun Yat-Sen University, Guangzhou, China; 4 Department of Ultrasound, Sun Yat-Sen Memorial Hospital, Sun Yat-Sen University, Guangzhou, China; 5 Department of Pathology, The First Affiliated Hospital, Zhengzhou University, Zhengzhou, China; 6 Cancer Research Institute, The First Affiliated Hospital, Hengyang Medical School, University of South China, Hengyang, Hunan, China; 7 Li Ka Shing Faculty of Medicine, Department of Pathology, The University of Hong Kong, Hong Kong, Guangdong, China

**Keywords:** SETD3, NUDT16, CHFR, PARylation, ubiquitination

## Abstract

Poly(ADP-ribosyl)ation (PARylation) is a critical posttranslational modification that plays a vital role in maintaining genomic stability *via* a variety of molecular mechanisms, including activation of replication stress and the DNA damage response. The nudix hydrolase NUDT16 was recently identified as a phosphodiesterase that is responsible for removing ADP-ribose units and that plays an important role in DNA repair. However, the roles of NUDT16 in coordinating replication stress and cell cycle progression remain elusive. Here, we report that SETD3, which is a member of the SET-domain containing protein (SETD) family, is a novel substrate for NUDT16, that its protein levels fluctuate during cell cycle progression, and that its stability is strictly regulated by NUDT16-mediated dePARylation. Moreover, our data indicated that the E3 ligase CHFR is responsible for the recognition and degradation of endogenous SETD3 in a PARP1-mediated PARylation-dependent manner. Mechanistically, we revealed that SETD3 associates with BRCA2 and promotes its recruitment to stalled replication fork and DNA damage sites upon replication stress or DNA double-strand breaks, respectively. Importantly, depletion of SETD3 in NUDT16-deficient cells did not further exacerbate DNA breaks or enhance the sensitivity of cancer cells to IR exposure, suggesting that the NUDT16-SETD3 pathway may play critical roles in the induction of tolerance to radiotherapy. Collectively, these data showed that NUDT16 functions as a key upstream regulator of SETD3 protein stability by reversing the ADP-ribosylation of SETD3, and NUDT16 participates in the resolution of replication stress and facilitates HR repair.

## Introduction

Protein posttranslational modifications (PTMs) involve the covalent addition of functional groups to specific amino acids in native proteins after their biosynthesis, and PTMs result in protein functional diversity (1). These modifications, including phosphorylation, ubiquitination, acetylation, methylation, SUMOylation, PARylation, and glycosylation, play crucial roles in almost all aspects of cell biological processes by modulating protein functions, protein‒protein interactions, cellular protein localization, and protein stability (2, 3). Emerging evidence has shown that PTM dysregulation frequently occurs and is involved in alterations in cellular functions and the development of multiple diseases, including human cancers (4, 5, 6, 7). Therefore, identifying novel regulatory PTM mechanisms that underlie cellular biological processes and tumorigenesis is important for understanding basic biological processes and cancer pathogenesis.

Poly(ADP-ribosyl)ation (PARylation) is a well-known PTM that participates in a variety of cellular processes, especially the DNA replication response and DNA repair; PARylation is mediated by distinct poly(ADP-ribose) polymerases (PARPs) that attach ADP-ribose moieties to substrates (8). Moreover, PARylation is a highly dynamic and reversible posttranslational modification that is rapidly hydrolyzed by dePARylation enzymes, including PARG, MacroD1, MacroD2, TARG1, ARH1, ARH3, NUDT5, NUDT9, NUDT16, and ENPP1 (9). PARG, which is the most abundant dePARylase, accounts for up to 90% of dePARylation activity and is primarily responsible for digesting glycosidic bonds between the ADP-ribose moieties of PAR chains, resulting in MARylation *via* the formation of free ADP-ribose unit linkages. The resulting MARylation is further attacked by linkage-dependent hydrolases, which completely remove the ADP-ribose moiety; these hydrolases include the ADP-ribose hydrolase family members ARH1 and ARH3 and the macrodomain-containing proteins MacroD1 and MacroD2 (10). Accumulating evidence has indicated that dePARylation also plays critical roles in a wide variety of cellular biological pathways, including the DNA damage response, immune response, and cell cycle checkpoints (8). For instance, PARG proteins can be recruited to sites of DNA damage through interactions with PCNA and can promote the repair of either DNA double-strand breaks (DSBs) or single-strand breaks (SSBs) (11). The nudix hydrolase NUDT5 can also be recruited to sites of DNA damage by binding to PARG, and it can regulate the supply and balance of cellular ATP levels in order to promote the recruitment of DNA repair proteins and homologous recombination repair (HR repair) (12). Gong *et al.* recently reported that another member of the Nudix hydrolase family, namely, NUDT16, interacts with PARylated 53BP1 and regulates its protein stability by recruiting the E3 ligase RNF146 to facilitate the ubiquitination and degradation of 53BP1; this study elucidated a novel mechanism by which 53BP1 stability is regulated mainly through NUDT16-mediated dePARylation (13). However, the role of dePARylation in the DNA damage response and the detailed mechanism by which these dePARylation enzyme’s function remain elusive.

SETD3, which is a member of both the SET-domain containing protein (SETD) family and the protein lysine methyltransferase (PKMT) family, has been reported to catalyze the addition of methyl groups to lysine residues in histone H3 through its conserved SET domain, and it plays important roles in cell development and differentiation (14). Recently, accumulating evidence has indicated that SETD3 performs a variety of functions that are involved in physiological and pathological processes, including tumorigenesis (15). For instance, SETD3 interacts with the transcription factors FoxM1 and p53 and promotes the expression of the downstream effectors VEGF, BAX, and PUMA, leading to the activation of VEGF-related oncogenesis and p53-induced apoptosis (14, 16). Moreover, SETD3 can directly methylate histone H3 at lysine 36 (H3K36) to activate oncogenic transcriptional programs and play vital roles in cell cycle regulation and cell death (17). Importantly, the protein level of SETD3 is cell cycle-dependent, peaking in the S phase, and both the GSK3β-mediated phosphorylation and FBXW7-dependent ubiquitination of SETD3 are necessary for its proteasome-mediated degradation, which then inhibits cell cycle progression and cell proliferation (18). Indeed, a line of clinical evidence further supports the notion that elevated SETD3 expression is closely correlated with high levels of cell proliferation and advanced-grade malignancy, suggesting that SETD3 protein levels might be considered a potential biomarker for predicting cancer progression and patient prognosis (19).

The breast cancer susceptibility gene 2 (BRCA2) is a potential tumor suppressor that has been widely studied for its ability to maintain genome stability through diverse processes, including homologous recombination (HR) repair and DNA replication stress. Structurally, BRCA2 is divided into multiple functional domains and motifs, including N-terminal DNA-binding domains (NTDs), which mediate interactions with PALB2; C-terminal DNA-binding domains (CTDs), which are capable of binding both ssDNA and dsDNA; and eight BRC repeats, which are responsible for the recruitment of RAD51 and DMC1; and a functionally distinct C-terminal RAD51 interaction motif (20). Mounting evidence has revealed that BRCA2 plays a fundamental role in HR and replication fork protection. On the one hand, BRCA2 stimulates the exchange of RPA and the loading of RAD51 recombinase on ssDNA by binding to BRCA1 and PALB2, resulting in the formation of RAD51 filaments on ssDNA and initiating homology template searches as well as strand exchange (21). On the other hand, BRCA2, together with FANCD2 and ABRO1, has an HR-independent function in stabilizing replication forks and protecting them against MRE11/EXO1-mediated degradation (22). Moreover, it has been documented that BRCA2 is required for the resolution of R-loops, a major endogenous source that mediates stalling of replication forks, in collaboration with the TREX-2 complex, suggesting that BRCA2 plays critical roles in resolving R-loop-associated transcription-replication conflicts (23). Although multiple binding partners of BRCA2 have been identified, the roles of this gene in modulating the DNA repair process and protecting against stalled replication forks have been elucidated, but the detailed mechanism governing BRCA2 recruitment to DSB sites and replication forks remains poorly understood.

In this study, we revealed a novel mechanism by which SETD3 protein stability is regulated by the PARylation-dependent ubiquitination-proteasome pathway and elucidated an undefined function of SETD3 in regulating replication stress and HR repair. First, we demonstrated that SETD3 forms a physical complex with BRCA2 at stalled replication fork and DSB sites, and positively regulates cell cycle progression and cell proliferation by reducing the R-loop-induced replication stress and enhancing HR repair in response to DNA double-strand breaks, thereby substantially increasing the resistance of cancer cells to IR exposure. Second, we observed that the protein levels of SETD3 and the nudix hydrolase NUDT16 fluctuate throughout the cell cycle and exhibit similar expression patterns that peak during the S phase. Mechanistically, we revealed that NUDT16 binds to PARylated SETD3 and reverses SETD3 PARylation, thereby impairing the recruitment of the E3 ligase CHFR and enhancing SETD3 protein stability. Importantly, depletion of endogenous SETD3 in NUDT16-deficient cells does not further exacerbate DNA breaks or enhance the sensitivity of cancer cells to IR treatment. Interestingly, the protein levels of both NUDT16 and SETD3 are significantly upregulated in IR-resistant cells, and SETD3 depletion greatly increased the proportion of apoptotic cells among IR-resistant cells after IR treatment. These data suggest that the NUDT16-SETD3 pathway may play critical roles in the induction of tolerance to radiotherapy and that targeting NUDT16 may be a promising strategy for reversing the resistance of cancer cells to IR therapy in clinical trials.

## Materials and Methods

### Cell culture and plasmid preparation

HEK293T, HeLa, BT549, MCF7, T47D, SUM149PT, SUM159PT, and MDA-MB-231 cells were purchased from American Type Culture Collection (ATCC) and cultured in Dulbecco’s modified Eagle’s medium (DMEM) supplemented with 10% FBS (Excell Corporation) and penicillin–streptomycin solution in an incubator at 37 °C in 5% CO2. MCF10A, HCC1806, HCC1937, MDA-MB-468, BT20, HS578T, and MDA-MB-436 cells were purchased from Procell Life Science & Technology Company and cultured with the provided cell culture medium. LacO-LacI reporter cells (U2OS-265) were kindly provided by Dr Roger Greenberg (University of Pennsylvania).

For plasmid construction, pDONR221 vectors containing the desired gene-coding sequences, including the SETD3, PARG, NUDT16, NUDT9, MacroD1, MacroD2, TARG1, ARH1, ARH3, ENPP1, TRIP12 and RNASEH1 gene-coding sequences, were obtained from GeneCopoeia Company. The entry vectors carrying these specific genes were then transferred to destination vectors with the indicated tags, such as the SFB and GFP tags, using the Gateway LR Clonase enzyme (Thermo Fisher) (24). SFB-PARP1, SFB-PARP2, SFB-PARP3, HA-ubiquitin, SFB-RNF146, and SFB-CHFR were previously described (25, 26, 27). HA-Ub-K48 and HA-Ub-K63 were kindly provided by Dr Ted Dawson (Addgene plasmid #17605, #17606). To construct the catalytically inactive SETD3 protein, the following primers were used: Y312A-Forward: GCTGGCACTCGATCCAACGCAG, Y312A-Reverse: AAAAATGTAAATCTGCTCTCC. Then, PCR-based mutation was performed according to the instructions of the kit (Takara). All the constructed plasmids were verified by DNA sequencing.

### Antibodies and reagents

A human anti-SETD3 (A304-071A-2), anti-BRCA2 (A303-434A), and anti-biotin antibodies (A150-109A) were purchased from Bethyl Laboratories. Human anti-NUDT16 (A17825), anti-HA (AE008), anti-TNKS2 (A16575), and anti-CHFR (A10447) antibodies were purchased from ABclonal. Human anti-ubiquitin (#3936) and anti-V5-Tag (D3H8Q) antibodies were purchased from Cell Signaling Technology. A human anti-PAR (4336-BPC-100) antibody was obtained from R&D Systems. Human anti-PARP1 (556494), anti-53BP1 (612522), and anti-AF555-γH2AX (560446) antibodies were purchased from BD Biosciences. Anti-Flag (66008-4-Ig) and anti-TNKS1 (18030-1-AP) antibodies were purchased from Proteintech Laboratories. A human anti-Rad51 (sc-8349) antibody was purchased from Santa Cruz. An anti-BrdU (ab6326) antibody was purchased from Abcam. An anti-IdU (TA190129) antibody was purchased from OriGene. Cycloheximide (CHX) and the proteasome inhibitor MG132 were purchased from Selleck Chemicals.

### DNA transfection and RNA interference

Plasmid transfections were performed using TurboFect reagent (Thermo Fisher) according to the manufacturer’s instructions. siRNA oligo transfection was performed utilizing Lipofectamine RNAiMAX reagent (Thermo Fisher) according to the manufacturer's protocol. The siRNA sequences targeting SETD3 were as follows: #1: CCAUGAAGGCCGAGGUCUUTT and #2: CCGGGAAUACUAUCGCCAATT; the NUDT16 siRNA sequence was CCACGACGAAUGCUAAGA.

### Lentivirus packaging and infection

Tet-pLKO-puro was a gift from Dmitri Wiederschain (Addgene plasmid # 21915). The shRNA sequences targeting SETD3 were as follows: #1: CCAUGAAGGCCGAGGUCUU and #2: CCGGGAAUACUAUCGCCAA and confirmed by DNA sequencing. Supernatants containing viral particles were collected 48 and 72 h after the co-transfection of lentiviral vectors and packaging plasmids (pMD2G and pSPAX2) into HEK293T cells. To generate stable cell lines, the indicated cells were incubated with viral-containing medium in the presence of 8 μg/ml polybrene for 12 h, and stable pools were selected with medium supplemented with 1 μg/ml puromycin for 3 days. The expression of the indicated genes was induced by the addition of 1 μg/ml doxycycline (Selleck) to the culture medium for 24 h.

### Proximity ligation assay

The indicated cells were pulse-labeled with 10 μM EdU for 15 min followed by treatment with 4 mM HU for another 3 h. After washing with PBS three times, the cells were pretreated with 0.5% Triton X-100 for 5 min at 4 °C followed by washing with PBS three times and then fixed with 3% formaldehyde at room temperature for 15 min, washed with PBS three times and blocked with 3% BSA at room temperature for 30 min. The resulting cells were subjected to Click-iT reaction to attach biotin to EdU and then incubated with the indicated primary antibodies at 4 °C overnight. Proximity ligation assays (PLA) were carried out by using a Duolink *In Situ* Red Starter Kit (DUO92101, Sigma-Aldrich) according to the manufacturer’s protocol. Images were acquired using Zeiss LSM 800 microscope and analyzed with ZEN Lite software. The data are representative of three independent experiments.

### RNA isolation and qRT‒PCR analysis

Total RNA was extracted from the indicated cells using TRIzol reagent (EZBioscience) and reverse transcribed into cDNA using a reverse transcription kit (EZBioscience). Quantitative polymerase chain reaction (qPCR) was performed using a Quantitative Real-time PCR Kit (EZBioscience) and a CFX96 Real-Time System (Bio-Rad). Relative mRNA expression was analyzed *via* comparative analysis of relative expression by the ^ΔΔ^Ct method and normalized using GAPDH as the internal control. The sequences of the qPCR primers specific for SETD3 were as follows: Forward: GAGTGGGAAGAGTATGTGCAGA; Reverse: TCAAAACCCTCGACAGAAGCC. The sequences of the qPCR primers specific for NUDT16 were as follows: Forward: CTGCGCTACGCCATACTGAT; Reverse: GTCAGACGCTTGGCATAGAAG.

### Cell cycle synchronization

Cells were synchronized at the G1/S phase boundary by HU (1 mM) treatment for 24 h and then released. The cells were cultured in fresh media and harvested at the indicated time points. The indicated cells were further analyzed by flow cytometry and Western blotting.

### Generation of knockout cells using CRISPR‒Cas9 technology

The following small guide RNAs (sgRNAs) were used to generate SETD3- and NUDT16-knockout cells: sgSETD3#1: TACAGCAACTGTGTCACCAA, sgSETD3#2: TACAGCAACTGTGTCACCAA, and sgNUDT16#1: AGGACAGAAGCCTAGAGGAC, and sgNUDT16#2: AGGACAGAAGCCTAGAGGAC. The synthetic sgRNAs were cloned into the pSB-CRISPR vector as previously described, followed by transfection into the indicated cells together with SB100 plasmids (24). Twenty-four hours after transfection, the indicated cells were cultured in the presence of puromycin (1 μg/ml), and puromycin-resistant cells were harvested and subjected to western blotting with the indicated antibodies.

### HR and NHEJ reporter assays

This assay was performed as previously described (28). Briefly, HeLa cells with stable integration of either the DR-GFP cassettes or EJ5-GFP cassettes were first transfected with SETD3 siRNAs for 24 h and then electroporated with 15 μg of the HA-tagged I-SceI plasmid using electric transfection equipment from Bio-Rad. The cells were collected at 48 h after transfection and subjected to flow cytometry analysis to quantify the ratio of GFP-positive cells. The means of three independent experiments are presented.

### Coimmunoprecipitation assay and Western blotting

Western blotting and Coimmunoprecipitation (Co-IP) were performed as previously described (27). Briefly, cells were lysed with NETN buffer containing a protease inhibitor cocktail (Bimake) and benzonase nuclease (Merck Millipore) at 4 °C for 30 min. Then, the cells were lysed, and the lysates were separated by SDS‒PAGE and analyzed with the indicated antibodies. For Co-IP with endogenous proteins, cell extracts were first incubated with anti-IgG antibodies or the indicated antibodies for 2 h at 4 °C, followed by incubation with protein A/G agarose (Thermo Fisher) with rotation at 4 °C overnight. For Co-IP with exogenous tagged proteins, supernatants were incubated with either V5 beads (Bimake), S beads (Merck Millipore), or HA beads (Bimake) and rotated at 4 °C overnight. After being centrifuged and washed with NETN buffer five times, the precipitates were analyzed by western blotting with the indicated antibodies.

### EdU-based assay of cell proliferation

The EdU assay was conducted according to the manufacturer’s instructions (Beyotime). Briefly, the indicated cells were harvested and seeded in a 6-well plate. After 1 h of incubation with 10 μM EdU, the cells were washed twice with PBS and fixed with 4% formaldehyde for 15 min at room temperature, followed by treatment with 0.5% Triton. Next, the harvested cells were subjected to click reaction using Alexa Fluor azide 488 reagents for 30 min, stained with DAPI, and visualized by fluorescence microscopy.

### DNA fiber analysis

The indicated cells were incubated with a prewarmed culture medium containing 25 μM IdU (Macklin, I811619) for 30 min, rapidly washed twice with prewarmed PBS, and incubated with 250 μM CldU (Sigma, C6891) for another 30 min. Next, the labeled cells were rapidly harvested and resuspended in ice-cold PBS at a concentration of 1 × 10^6^ cells per milliliter, daubed on glass slides, and lysed with 8 μl of spreading buffer (50 mM EDTA, 0.5% SDS, 200 mM Tris-HCl) for 5 min. Then, the glass slides were tilted to extend the labeled DNA. For fixation, glass slides were immersed in methanol/acetic acid (3:1) for 30 min. After washing twice with PBS, the glass slides were incubated in 2.5 M HCl for 2 h at room temperature to denature the DNA molecules. Then, the glass slides were treated with 5% BSA blocking buffer for 30 min and treated with anti-IdU (OriGene, TA190129, 1:200) and anti-CldU (Abcam, ab2326, 1:500) at 4 °C overnight. After the overnight incubation, the slides were washed twice with PBST followed by staining with Alexa Fluor 488 goat anti-mouse (Thermo Fisher, A11001, 1:2000) and Alexa Fluor 555 goat anti-rat (Thermo Fisher, A21434, 1:2000) antibodies. After being mounted, the glass slides were imaged with a Zeiss LSM 800 microscope, and DNA fiber lengths were analyzed using ImageJ software.

### Cell survival assay

A cell survival assay was performed to assess the ability of single cells to form colonies *in vitro* after exposure to irradiation. Briefly, the indicated cells were seeded in twelve-well plates (500 cells/well) for 7 days and then subjected to IR treatment at the indicated doses followed by the addition of 1 μg/ml doxycycline (Selleck) to the culture medium to induce the expression of the indicated shRNA. After being cultured for another 7 days, the cell clones were fixed with 4% formaldehyde and treated with crystal violet solution for 30 min. Colonies containing more than 50 cells were defined as positive survivors and were counted using ImageJ software.

### Alkaline comet assay

Cells were harvested at the indicated time points after IR treatment, mixed with molten low-melting-point agarose, and immediately dropped onto a slide. After the agarose had gelled, the slides were incubated in a covered dish containing alkaline lysis solution (1.2 M NaCl, 100 mM Na_2_EDTA, 0.1% sodium lauroyl sarcosinate, 0.26 M NaOH) overnight at 4 °C in the dark. After overnight lysis, the slides were submerged in room temperature alkaline wash buffer (0.03 M NaOH, 2 mM Na_2_EDTA) for 30 min. After three rinses, the slides were incubated in 1 × alkaline electrophoresis buffer at 25 V for 30 min. Then, the slides were rinsed and neutralized in distilled water, stained with SYBR green dyes (Thermo Fisher) for 10 min, and further imaged with a fluorescence microscope. Comet parameters were automatically analyzed and quantified using the Comet Assay Software Project (CASP).

### *In vivo* ubiquitination assay

This procedure was performed as previously described (27). Briefly, the indicated cells were transfected with the indicated plasmids for 24 h, and then, the proteasome inhibitor MG132 (10 μM) was added, and the cells were incubated for another 4 h before harvesting. The clarified cell lysates were incubated with anti-S protein beads for coimmunoprecipitation assay, and the immunoprecipitated proteins were subjected to western blotting analysis using the indicated antibodies.

### Statistical analysis

All the experiments were repeated at least three times, and the results are presented as the mean ± SD and were analyzed using GraphPad Prism 9 software. The statistical significance of the data was assessed by one-way ANOVA. Significance is indicated by asterisks (∗∗∗∗*p* < 0.0001; ∗∗∗*p* < 0.001; ∗∗*p* < 0.01; ∗*p* < 0.05; ns, not significant), and *p* <0.05 was considered statistically significant.

## Results

### SETD3 regulates cell proliferation and promotes radioresistance in cancer cells

**Figure 1:**
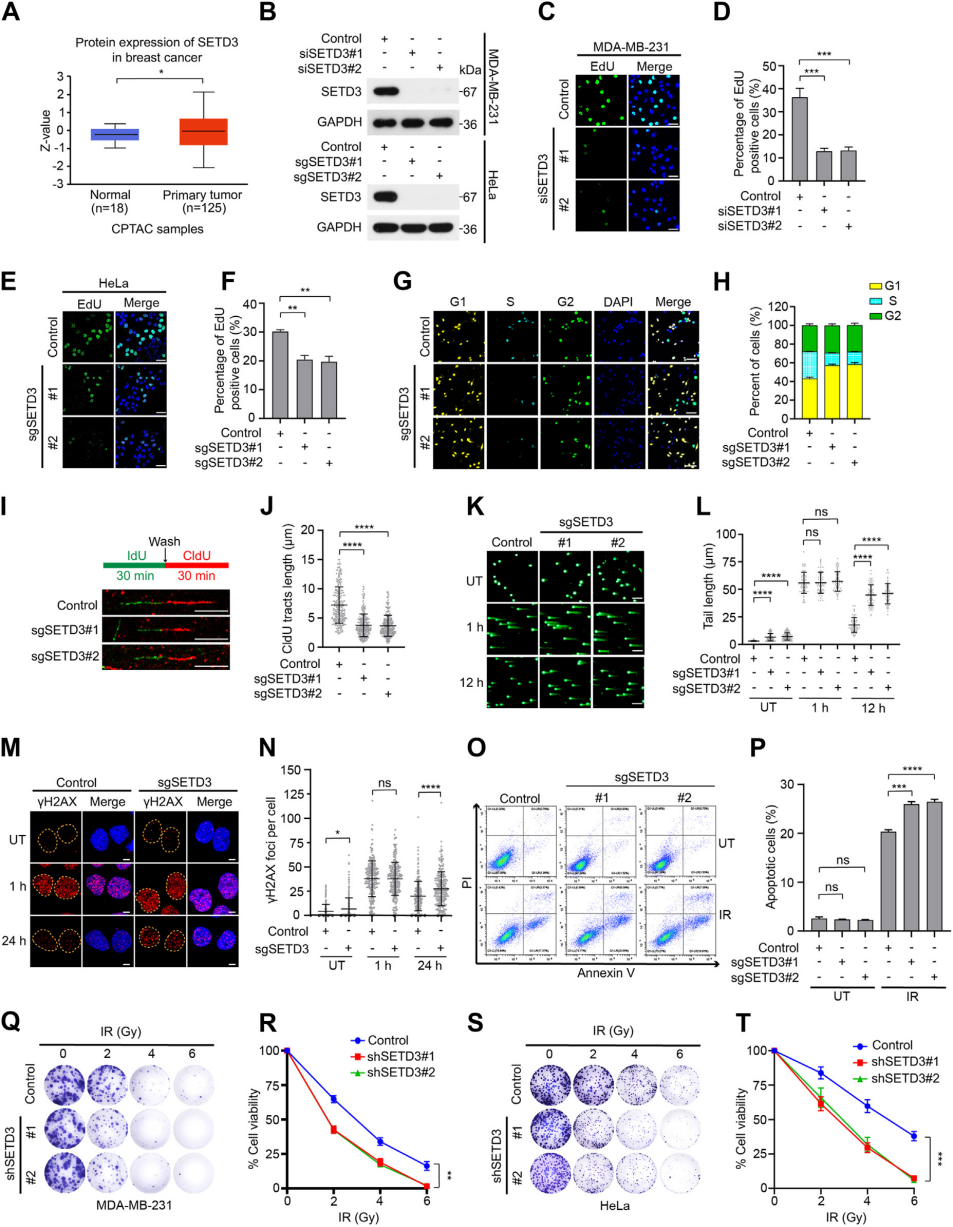
**SETD3 depletion suppresses cell proliferation and promotes the radiosensitivity of cancer cells.***A*, analysis of SETD3 protein levels in the Clinical Proteomic Tumor Analysis Consortium (CPTAC) breast cancer dataset (http://ualcan.path.uab.edu/index.html). *B*, *upper panel*: MDA-MB-231 cells were transfected with the indicated SETD3 siRNAs for 48 h, and protein levels were examined by western blotting analysis with the indicated antibodies. *Lower panel*: The indicated sgRNAs targeting endogenous SETD3 were transfected into HeLa cells, and the cell lysates were analyzed by western blotting assay with the indicated antibodies. *C*–*F*, SETD3 depletion significantly inhibited S phase progression in both MDA-MB-231 and HeLa cells. SETD3-knockdown cells (MDA-MB-231) or SETD3-knockout cells (HeLa) were incubated with EdU (10 μM) for 1 h followed by EdU-click assay with Alexa Fluor 488 azide staining (n = 3). Representative images of EdU-positive cells are shown in (*C*) and (*E*). Scale bar, 30 μm. The percentage of EdU-positive cells was then quantified (*D* and *F*). Data represent the mean ± SD from three independent experiments. ∗∗∗*p* < 0.001; ∗∗*p* < 0.01; ∗*p* < 0.05; ns, not significant. *G* and *H*, HeLa cells with stable integration of Clover-Geninin and mKO2-Cdt elements were transfected with the indicated sgRNAs targeting endogenous SETD3, Immunofluorescence analyses, and representative FUCCI images are shown in (*G*). Scale bar, 40 μm. The percentage of cells in the G1, S, and G2 phases was quantified (*H*). *I* and *J*, depletion of endogenous SETD3 severely impaired normal DNA replication. Representative images of CldU and IdU replication tracks are shown in (*I*), and the CldU tract length was measured in both control cells and SETD3-deficient cells. At least 300 tracks were measured in each group (*J*). Scale bar, 5 μm. (*K* and *L*) SETD3 depletion inhibits DNA repair. Control or SETD3-deficient HeLa cells were either untreated or treated with IR (4 Gy) and allowed to recover for the indicated times, and then, alkaline comet assays were conducted (*K*). Scale bar, 40 μm. The levels of DNA breakage were determined by measuring the length of the comet tail area. The comet tail moment was quantified using CASP software. At least 60 comet tails were analyzed in each group (*L*). *M* and *N*, SETD3 depletion prolongs γ-H2AX foci appearance. Control or SETD3-deficient HeLa cells were either untreated or treated with IR (4 Gy) and allowed to recover for the indicated times followed by γ-H2AX immunofluorescence. Representative images of γ-H2AX foci are shown in M. Scale bar, 5 μm. *N*, γ-H2AX foci in each group were quantified using ImageJ software, and quantification results are presented as the mean ± SD. More than 100 cells were counted in each experiment. *O* and *P*, depletion of SETD3 resulted in an increased percentage of apoptotic cells after IR exposure. Control or SETD3-deficient HeLa cells were either untreated or treated with IR (10 Gy) and then allowed to recover for 72 h followed by Annexin V/PI staining and flow cytometry analysis (*O*). The results of apoptotic cell quantification in both groups are shown as the mean ± SD (n = 3). *Q*–*T*, depletion of SETD3 greatly enhanced the sensitivity of both HeLa and MDA-MB-231 cells to IR treatment. HeLa and MDA-MB-231 cell lines stably expressing SETD3 shRNA (Tet-shSETD3) were generated. The resulting cells were first irradiated at different doses as indicated and subjected to a cell survival assay (*Q* and *S*). Experiments were performed in triplicate, and the resulting colonies were quantified using ImageJ software.

To evaluate the clinical significance of SETD3 in breast cancer, we first analyzed the protein levels of SETD3 using the Clinical Proteomic Tumor Analysis Consortium (CPTAC) (29). As shown in Figure 1*A*, the protein levels of SETD3 were upregulated in breast cancer tissues compared with those in normal breast tissue. Indeed, SETD3 protein expression was markedly increased in both estrogen receptor (ER) positive and triple-negative breast cancer (TNBC) cells relative to normal breast epithelial MCF10A cells and human epidermal growth factor receptor 2 (HER2) positive SKBR3 cells (Fig. S1*A*). Additionally, analyses based on The Cancer Genome Atlas (TCGA) database showed that for only patients with triple-negative breast cancer (TNBC), patients with low levels of SETD3 had a more favorable prognosis than those with high SETD3 expression (Fig. S1, *B*–*F*), suggesting that SETD3 might be a valuable prognostic marker for TNBC patients. Next, we explored whether SETD3 plays oncogenic roles in breast cancer, and an EdU incorporation assay and FUCCI system were utilized (30). As shown in Figure 1, *B*–*F*, depletion of endogenous SETD3 significantly decreased the proportion of MDA-MB-231 cells and HeLa cells in the S phase. Furthermore, we generated fluorescent HeLa cells to monitor specific cell phases by simultaneously integrating mKO2-Cdt1 and Clover-Geminin elements (30). Knockout of endogenous SETD3 greatly increased the percentage of cells in the G1 phase and decreased the proportion of cells in the S phase (Fig. 1, *G* and *H*). In addition, we synchronized both wild-type and SETD3-deficient HeLa cells at the G1/S phase boundary and then released them to examine the progression of the indicated cells to the S phase. Indeed, depletion of endogenous SETD3 led to a dramatic delay in S phase progression relative to that in wild-type HeLa cells (Fig. S2*A*). Given that proper DNA synthesis and replication are prerequisites for S phase progression, we wondered whether SETD3 plays vital roles in DNA replication, and a DNA fiber assay was performed. As shown in Figure 1, *I* and *J*, depletion of endogenous SETD3 extensively slowed the rate of nascent DNA synthesis, indicating that SETD3 is required for proper DNA synthesis. Taken together, these results strongly support the notion that SETD3 accelerates S phase progression and promotes cell proliferation.

Since previous studies have shown that SETD3 regulates the p53-dependent induction of apoptosis in response to DNA damage (14), we wondered whether SETD3 plays essential roles in the DNA damage response. To this end, we first performed an alkaline comet assay and found that knocking out endogenous SETD3 significantly increased the length and DNA content of comet tails (Fig. 1, *K* and *L*). To further confirm these observations, an immunofluorescence assay was performed to determine the fluorescence intensity of γH2AX, which is a marker of DNA double-strand breaks. Consistently, depletion of SETD3 resulted in elevated levels of spontaneous γH2AX foci and IR-induced γH2AX foci 24 h after IR exposure (Fig. 1, *M* and *N*). To determine the biological importance of SETD3 in the response to DNA damage, Annexin V/PI staining and flow cytometry were performed. As shown in Figure 1, *O* and *P*, the depletion of endogenous SETD3 profoundly increased the proportion of apoptotic cells after IR treatment. Consistent with these findings, SETD3 depletion dramatically enhanced cellular sensitivity to radiation compared with that of control cells (Figs. 1, *Q*–*T* and S1*L*). To further determine whether SETD3 expression is closely associated with cell proliferation and radioresistance, we analyzed the percentage of SETD3-overexpressing MCF10A cells in the S phase and the sensitivity of these cells towards IR treatment. Compared with those in the control group, cells overexpressing SETD3 displayed a marked increase in cell proliferation capability and resistance to IR exposure (Fig. S1, *G*–*K*). Collectively, these findings support the notion that SETD3 protein expression is associated with cell proliferation and radiation resistance.

### SETD3 promotes replication fork stability by associating with BRCA2

**Figure 2:**
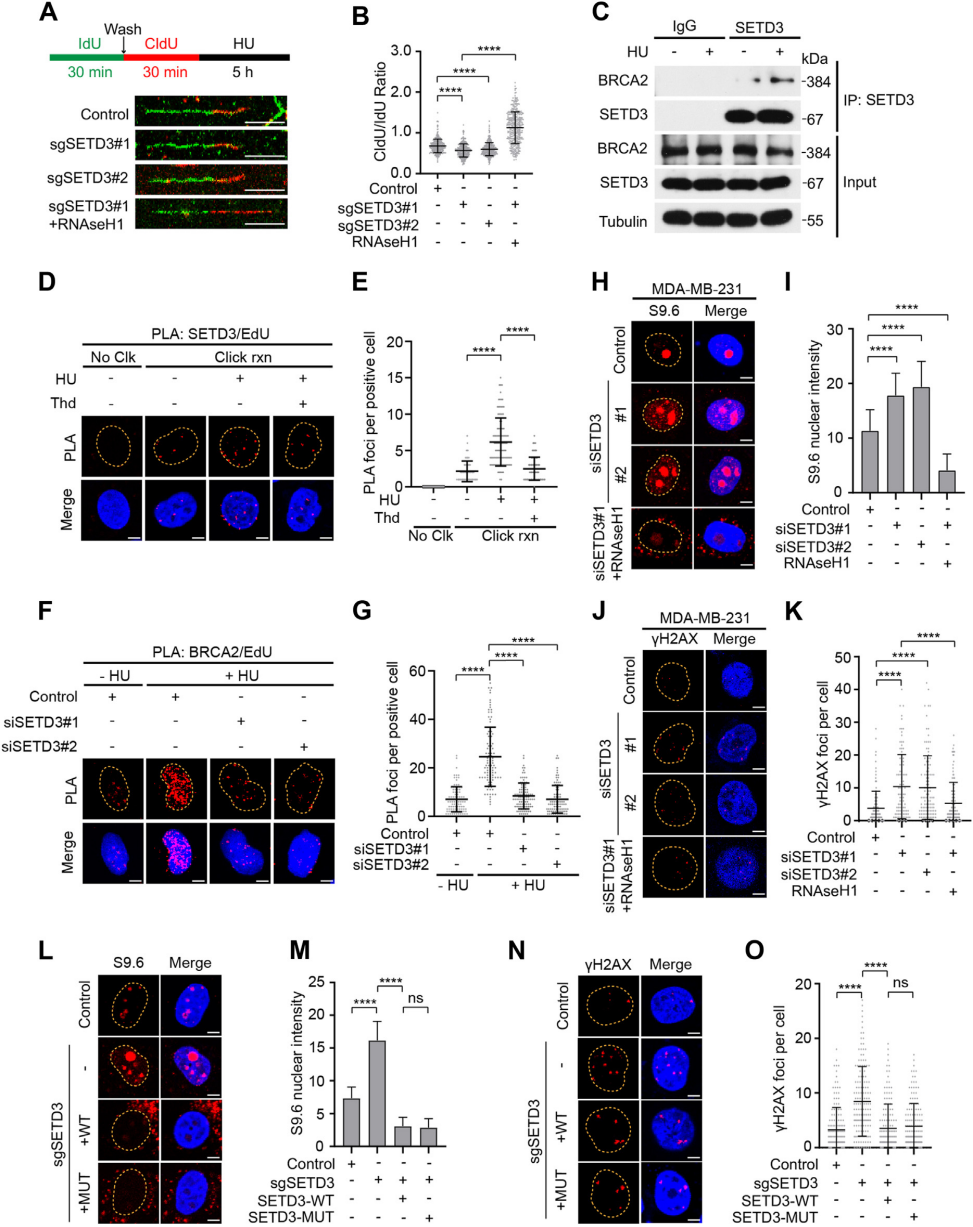
**Loss of SETD3 triggers R-loop accumulation and promotes R-loop-dependent DNA damage.***A* and *B*, depletion of endogenous SETD3 markedly enhanced the degradation of stalled replication forks. Representative images of CldU and IdU replication tracks are shown in (*A*), and the ratio of CldU to IdU track lengths was calculated in both control cells and SETD3-deficient cells. Scale bar, 5 μm. Plots show the average ratio of CldU:IdU tract lengths from three independent assays, and at least 300 tracks were measured in each group (*B*). *C*, HeLa cells were first treated with 4 mM HU for 3 h, and the cell lysates were harvested and subjected to a Co-IP assay using anti-SETD3 antibodies followed by western blotting analysis with the indicated antibodies. *D* and *E*, analysis of SETD3 recruitment by PLA. HeLa cells were pulse-labeled with 10 μM EdU for 15 min followed with or without 10 μM thymidine chase for 1 h (Thd). Cells were then left untreated or treated with 4 mM HU for 3 h and subjected to PLA with anti-SETD3 and anti-biotin antibodies. Scale bar, 5 μm. *E*, quantification of SETD3/EdU PLA foci number per focus positive cell. At least 100 cells were counted in each individual experiment. *F* and *G*, SETD3 knockdown significantly reduces BRCA2/EdU PLA foci formation. HeLa cells were transfected with the indicated siRNAs for 48 h and then pulse-labeled with 10 μM EdU for 15 min followed with or without 4 mM HU for 3 h and subjected to PLA with anti-BRCA2 and anti-biotin antibodies. Scale bar, 5 μm. *G*, quantification of BRCA2/EdU PLA foci number per focus positive cell. At least 100 cells were counted in each individual experiment. *H* and *I*, depletion of SETD3 led to a substantial increase in nucleolar S9.6 signals. Both control and indicated MDA-MB-231 cells were subjected to immunofluorescence assay using anti-S9.6 antibodies, and a representative image is shown in (*H*). Scale bar, 5 μm. The nuclear S9.6 signal intensity was quantified by ImageJ software, and at least 100 cells were analyzed (*I*). *J* and *K*, depletion of SETD3 resulted in the accumulation of γH2AX foci in MDA-MB-231 cells. Representative micrographs showing the immunofluorescence assay results of SETD3-deficient cells are shown in (*J*). Scale bar, 5 μm. Quantification of the γH2AX foci per cell using ImageJ software is shown in (*J* and *K*), and at least 100 cells were analyzed. *L* and *M*, HeLa control cells or SETD3-deficient cells transfected with either SETD3 wild-type or catalytically inactive constructs were subjected to immunofluorescence assay with anti-S9.6 antibodies, and representative images of S9.6 signal intensity are shown in L. Scale bar, 5 μm. The nuclear S9.6 signal intensity was quantified by ImageJ software, and at least 100 cells were analyzed (*M*). *N* and *O*, HeLa control cells or SETD3-deficient cells transfected with either SETD3 wild-type or catalytically inactive constructs were subjected to immunofluorescence assay with anti-γH2AX antibodies, and representative images of γH2AX foci are shown in N. Scale bar, 5 μm. The γH2AX foci were quantified by ImageJ software, and at least 100 cells were analyzed (*O*).

Previous studies have indicated that replication fork stability contributes to the maintenance of genome stability and resistance to DNA-damaging agents independently of homologous recombination activities that are mediated by DSBs (31, 32). Recently, a bimolecular fluorescence-based screen revealed that SETD3 functions as a novel binding partner for PCNA (33), which raises the possibility that SETD3 might be able to regulate replication fork stability. Indeed, depletion of endogenous SETD3 significantly promoted nascent DNA degradation after treatment with HU (Figs. 2, *A* and *B* and S3, *A* and *B*). To clarify the detailed mechanism of SETD3 in maintaining replication fork stability, we utilized anti-SETD3 antibodies to pulldown its interacting partners and the purified proteins were subjected to mass spectrometry analysis to identify potential SETD3-binding partners. As indicated in Figure 2*C*, a complex containing SETD3/BRCA2 was readily detected (Table S1 and Fig. S3*H*). Moreover, the association between SETD3 and BRCA2 was obviously enhanced by HU treatment (Fig. 2*C*). It has been well documented that BRCA2 can accumulate at stalled replication forks and protect stalled replication forks from nucleolytic degradation (34), and prevent R-loop accumulation to alleviate replication stress (23). We wondered whether SETD3 can also be recruited to stalled replication forks, and an in-situ proximity ligation assay (PLA) was performed. As shown in Figure 2, *D* and *E*, cells treated with HU exhibited a dramatic increase in the number of SETD3/biotin PLA foci. Interestingly, SETD3 also slightly accumulated on mature DNA (after 1 h of thymidine chase) after HU treatment (Fig. 2, *D* and *E*), indicating that SETD3 may also play a critical role in DNA replication under unperturbed conditions. To determine whether the interaction of SETD3 with BRCA2 is important for the enrichment of BRCA2 at the replication fork, we depleted endogenous SETD3 with two individual siRNAs and used the PLA assay to measure BRCA2/biotin PLA focus formation. As indicated in Figure 2, *F* and *G*, the depletion of SETD3 dramatically decreased the number of BRCA2/biotin PLA foci, suggesting that SETD3 is required for BRCA2 localization to stalled replication forks. Furthermore, the depletion of endogenous SETD3 did not affect BRCA2 protein stability (Fig. S3*C*).

To further confirm the role of SETD3 in resolving R-loops, which are major obstacles to replication, we utilized S9.6 antibodies to measure the endogenous levels of R-loops, and immunofluorescence was performed. As shown in Figure 2, *H* and *I* and Fig. S3, *D* and *E*, depletion of SETD3 resulted in a marked increase in the nucleolar S9.6 signal in both HeLa cells and MDA-MB-231 cells. Furthermore, reintroduction of the RNA endonuclease RNAseH1, which is an enzyme that is responsible for the degradation of RNA‒DNA hybrids (R-loops) (35), into SETD3-deficient cells completely reversed the nucleolar enrichment of R-loops and the CldU track length after HU treatment (Fig. 2, *A*, *B*, *H*, and *I*), indicating that SETD3 plays vital roles in protecting the nascent strand at stalled replication forks that are induced by R-loops.

Growing evidence has indicated that R-loops not only function as a major source of replication stress but also as a key cause of endogenous DNA double-strand breaks (36). Therefore, we sought to determine whether SETD3 depletion has any effect on endogenous γ-H2AX foci formation. As shown in Figure 2, *J* and *K* and Fig. S3, *F* and *G*, depletion of SETD3 resulted in a substantial increase in the number of γH2AX foci in both HeLa and MDA-MB-231 cells. Consistently, the reintroduction of RNAseH1 constructs into SETD3-deficient cells nearly abolished the formation of γH2AX foci induced by SETD3 depletion (Fig. 2, *J* and *K*), further indicating that SETD3 is also important for repairing endogenous DSBs. To determine whether SETD3 methyltransferase activity is involved in R-loop resolution and γH2AX foci formation, we generated a catalytically inactive mutant (Y312A) of SETD3, and S9.6 antibody staining was performed (37). As shown in Figure 2, *L* and *M*, the reconstitution of SETD3-deficient cells with either wild-type SETD3 or the SETD3 mutant (Y312A) effectively restored the intensity of the S9.6 signal. Consistent with these results, re-expression of both wild-type SETD3 and the SETD3 mutant in SETD3-deficient cells reversed the change in γH2AX foci magnitude caused by SETD3 depletion with similar efficiency (Fig. 2, *N* and *O*), demonstrating that the catalytic activity of SETD3 is not essential for the SETD3-mediated resolution of R-loops.

### SETD3 promotes homologous recombination repair

**Figure 3:**
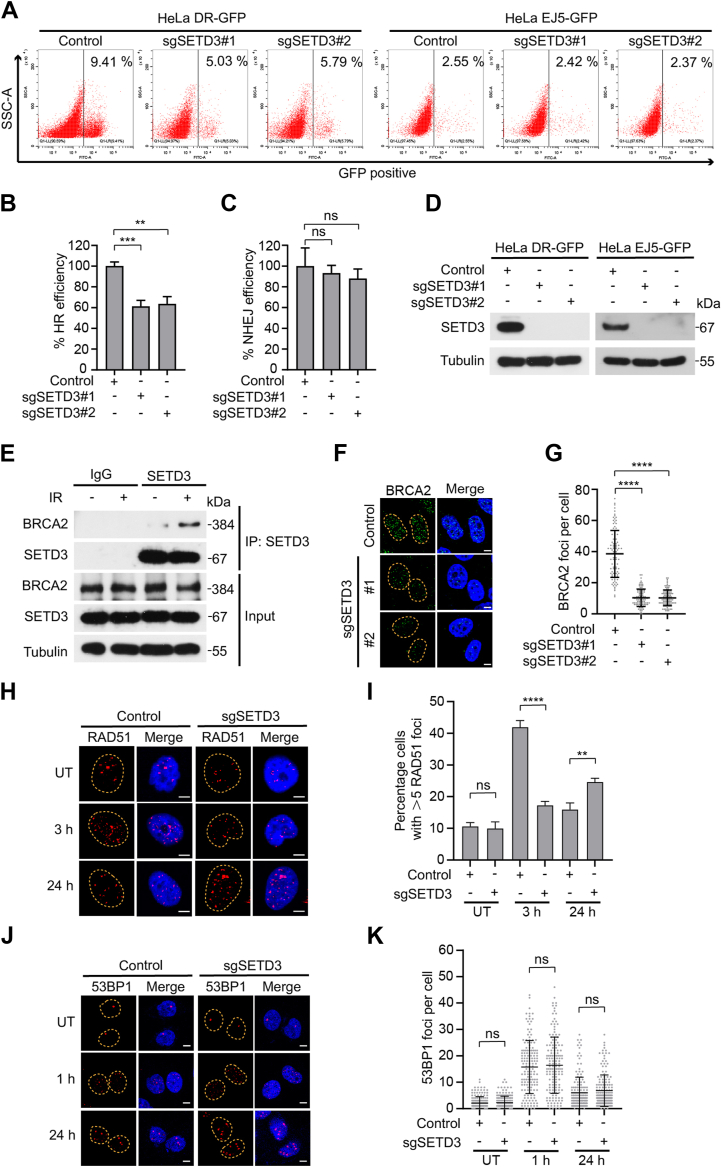
**Loss of SETD3 suppresses BRCA2-mediated HR repair.***A*–*D*, depletion of SETD3 suppresses the efficiency of HR. HeLa cells carrying DR-GFP or EJ5-GFP cassettes were transfected with the indicated siRNAs targeting endogenous SETD3. I-SecI transfection for 48 h, and then, the harvested cells were subjected to flow cytometry analysis and Western blot assay (*A* and *D*). GFP-positive cells were quantified and normalized as indicated in (*B* and *C*). *E*, HeLa cells were first left untreated or treated with IR (10 Gy) for 2 h, and the cell lysates were harvested and subjected to a Co-IP assay using anti-SETD3 antibodies followed by western blotting analysis with the indicated antibodies. *F* and *G*, depletion of SETD3 significantly inhibited BRCA2 foci formation. HeLa control and SETD3-deficient cells were subjected to 10 Gy of irradiation and allowed to recover for 2 h, followed by BRCA2 immunostaining. Representative images of BRCA2 foci are shown in (*F*). Scale bar, 5 μm. *G*, BRCA2 foci in each group were quantified using ImageJ, and statistical results are shown as the mean ± SD. More than 100 cells were counted in each experiment. *H*, depletion of SETD3 inhibited endogenous RAD51 foci formation and extended the duration of their appearance. HeLa control and SETD3-deficient cells were subjected to 4 Gy of irradiation and allowed to recover for the indicated times, followed by RAD51 immunostaining. Representative images of RAD51 foci are shown in (*H*). Scale bar, 5 μm. *I*, RAD51 foci in each group were analyzed using ImageJ, and statistical results are shown as the mean ± SD. More than 100 cells were counted in each experiment. *J* and *K*, depletion of SETD3 had no effect on 53BP1 foci formation. HeLa control and SETD3-deficient cells were subjected to 4 Gy irradiation and then allowed to recover for the indicated times. Representative images of 53BP1 foci are shown in (*J*). Scale bar, 5 μm. *K*, 53BP1 foci in each group were quantified using ImageJ software, and the results are shown as the mean ± SD. More than 100 cells were counted in each experiment.

Previous studies have suggested that SETD3 is an important regulator of the DNA damage response (14)^.^ To explore the detailed molecular mechanism by which SETD3 participates in DNA damage repair, the DR-GFP and EJ5-GFP reporter systems were used to examine the efficiency of HR and NHEJ (28, 38). As shown in Figure 3, *A*–*D*, SETD3 depletion *via* two individual sgRNAs greatly decreased the frequency of HR repair but did not affect NHEJ efficiency. Given that BRCA2 forms a complex with RAD51 to stabilize the formation of RAD51 filaments and facilitates homologous sequence pairing and DNA strand exchange (39), we speculated that SETD3-mediated HR repair may largely rely on its physical interaction with BRCA2. In line with our previous findings (Fig. 2*C*), endogenous BRCA2 was readily immunoprecipitated with the SETD3 antibody (Fig. 3*E*). Moreover, the interaction between SETD3 and BRCA2 was obviously enhanced following IR exposure (Fig. 3*E*). To determine the physiological function of the interaction between SETD3 and BRCA2, we examined the capabilities of BRCA2 foci formation. Depletion of endogenous SETD3 led to a dramatic decrease in the number of BRCA2 IRIF formation (Fig. 3, *F* and *G*), suggesting that SETD3 plays an important role in the recruitment of BRCA2 to sites of DNA damage. To further verify the critical roles of SETD3 in the regulation of the HR repair process, we detected the recruitment of the HR-related protein RAD51 and the NHEJ core factor 53BP1 to sites of DNA damage (40, 41). Consistently, depletion of SETD3 significantly decreased the early recruitment of RAD51 to IR-induced DNA damage foci and greatly increased the retention of RAD51 foci at the late stage of IR-induced DSBs (Fig. 3, *H* and *I*). However, SETD3 depletion did not affect the recruitment and retention of endogenous 53BP1 (Fig. 3, *J* and *K*). Collectively, these results suggest that SETD3 promotes HR repair in a BRCA2-dependent manner.

### NUDT16 interacts with PARylated SETD3

**Figure 4:**
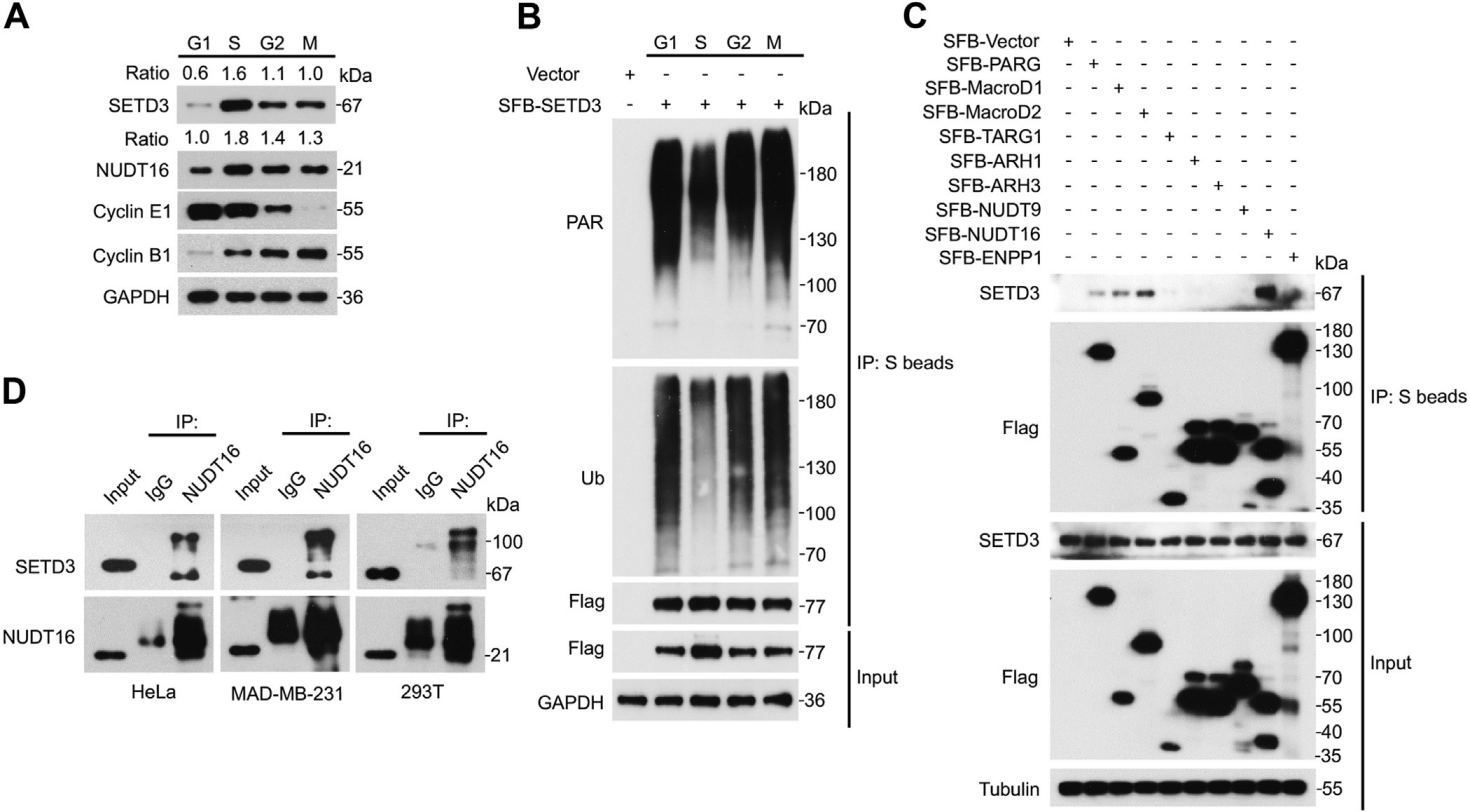
**NUDT16 interacts with PARylated SETD3.***A*, HeLa cells were synchronized at the G1/S phase boundary by HU (1 mM) treatment for 24 h and then released. The cell lysates were harvested at the indicated time points after release and subjected to western blotting analysis. Cyclin E1 is mainly expressed during the late G1 phase until the end of the S phase. Cyclin B1 is a key regulator of mitotic entry, and its protein levels peak at M phase. *B*, HeLa cells were first transfected with SFB-SETD3 for 24 h followed by synchronization at different stages as indicated above, and then, the cell lysates were harvested and subjected to a Co-IP assay using anti-S-beads followed by western blotting analysis with the indicated antibodies. *C*, the indicated plasmids were transiently transfected into HeLa cells for 24 h, and the cell lysates were harvested and subjected to Co-IP analysis using anti-S beads followed by western blotting analysis with the indicated antibodies. *D*, the indicated cells were harvested, and the cell lysates were subjected to a Co-IP assay using anti-IgG or anti-NUDT16 antibodies followed by Western blotting analysis.

Previous studies have indicated that SETD3 protein levels are cell cycle-dependent and are strictly regulated by GSK3β-mediated phosphorylation *via* the ubiquitin‒proteasome pathway (18, 42). Consistent with previous findings, endogenous SETD3 protein levels peaked in the S phase and then declined in the G2 phase (Figs. 4*A* and S4*A*). Nevertheless, quantitative real-time PCR results showed that the mRNA levels of SETD3 were not obviously altered throughout the entire cell cycle (Fig. S4*B*), suggesting that SETD3 protein levels fluctuate at the posttranslational level rather than at the posttranscriptional level during cell cycle progression.

PARP-mediated PARylation plays vital roles in modulating protein stability upon exposure to distinct stresses (26, 27, 43) Therefore, we explored whether SETD3 protein stability is regulated by the PARylation-dependent ubiquitin proteasomal pathway. As shown in Figure 4*B*, the PARylation levels of endogenous SETD3 were low in the S phase, gradually increased in the G2 phase, and peaked in the M phase. Importantly, the level of ubiquitinated SETD3 exhibited a similar pattern to that of PARylated SETD3, suggesting that PARylation of SETD3 is important for its ubiquitination (Fig. 4*B*). Given that PARylation has been identified as a transient and reversible posttranslational modification and that it is quickly removed by dePARylation enzymes, including PARG, MacroD1, MacroD2, TARG1, ARH1, ARH3, NUDT9, NUDT16, and ENPP1 (8, 9), we sought to determine which dePARylation enzyme is responsible for removing PARylation from SETD3 during the S phase; thus, the indicated plasmids were transiently transfected and Co-IPs were performed. As shown in Figure 4*C*, the dePARylation enzyme NUDT16 showed a strong binding affinity for endogenous SETD3, whereas PARG, MacroD1, and MacroD2 exhibited relatively weak binding to SETD3. To further confirm the interaction between endogenous SETD3 and NUDT16, different cell lysates were harvested and subjected to Co-IP assays. As shown in Figure 4*D*, both endogenous SETD3 and NUDT16 were readily detected in the immunoprecipitated complex. Notably, NUDT16 is mainly responsible for digesting both MAR and PAR chains (44), indicating that NUDT16 could bind to MARylated SETD3 with a relatively low molecular weight as well as PARylated SETD3 with a high molecular weight, as indicated by the shifted bands. Moreover, the expression pattern of the NUDT16 protein during cell cycle progression was closely related to that of the SETD3 protein, with the highest levels occurring in the S phase (Fig. 4*A*). Taken together, these results firmly indicate that the dePARylation enzyme NUDT16 binds to and forms a complex with PARylated SETD3, resulting in a decrease in the levels of endogenous PARylated SETD3.

### NUDT16 removes SETD3 PARylation and enhances SETD3 protein stability

**Figure 5:**
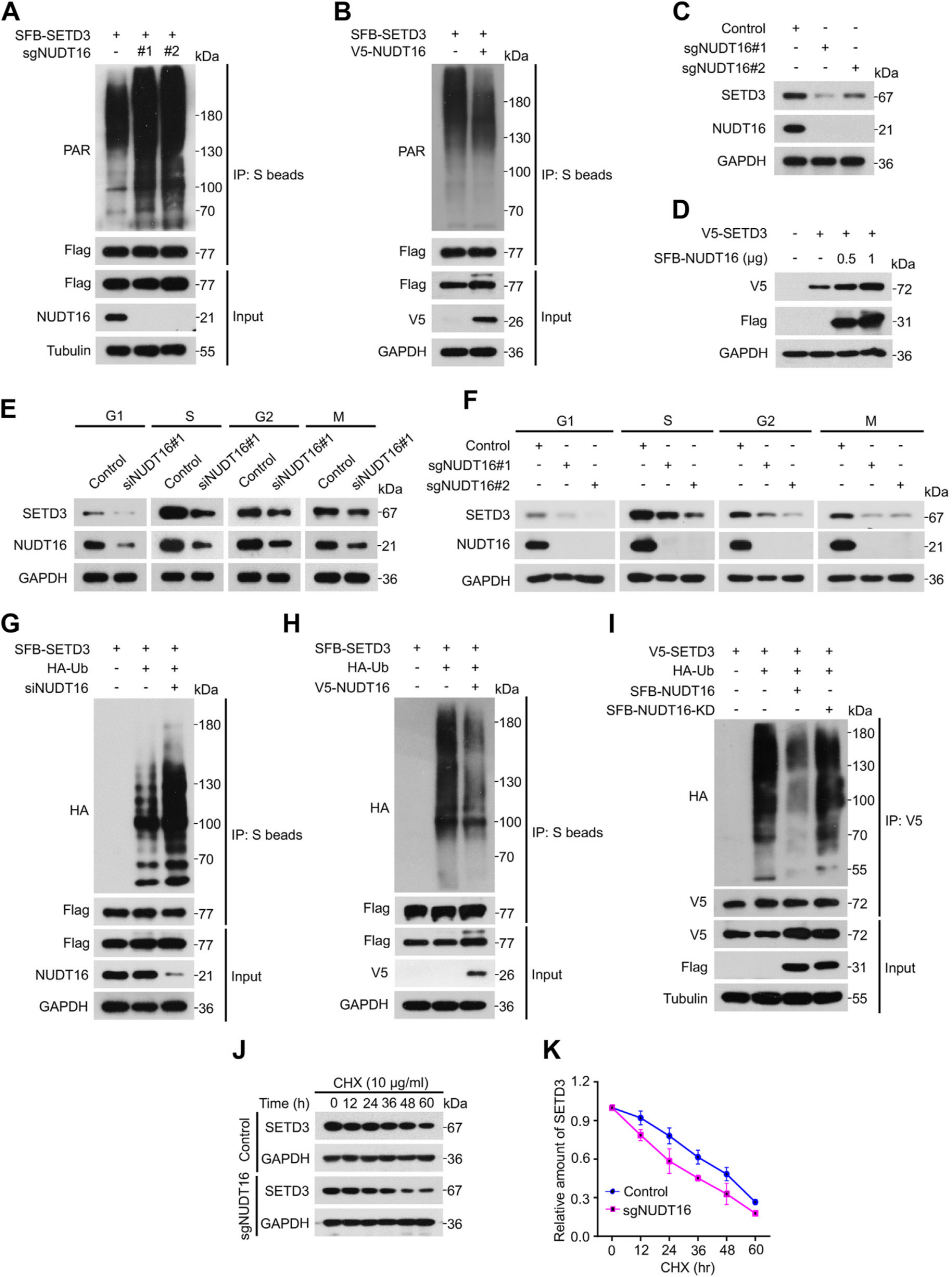
**NUDT16 mediates the dePARylation of SETD3 and enhances its protein stability.***A* and *B*, NUDT16 reverses the PARylation of the SETD3 protein. NUDT16 wild-type and knockout HeLa cells were transfected with SFB-SETD3 for 24 h followed by the addition of MG132 (10 μM) for another 6 h, and the cell lysates were subjected to Co-IP using anti-S beads followed by western blotting with the indicated antibodies (*A*). HeLa cells were cotransfected with the indicated plasmids for 24 h, and the cell lysates were harvested and subjected to Co-IP analysis using S beads followed by western blotting analysis with the indicated antibodies (*B*). *C* and *F*, NUDT16 positively regulates SETD3 expression. NUDT16-knockout HeLa cells were lysed and then analyzed by western blotting with the indicated antibodies (*C*). HeLa cells were cotransfected with the indicated plasmids for 24 h. Then, the cells were lysed and subjected to western blotting analysis (*D*). Control and NUDT16-depleted HeLa cells were synchronized at the G1/S boundary by HU (1 mM) treatment for 24 h and released at the indicated times. The cell lysates were harvested and subjected to western blotting analysis with the indicated antibodies (*E*). NUDT16-knockout HeLa cells were synchronized at the G1/S boundary by HU (1 mM) treatment for 24 h and then released for the indicated times. The cell lysates were harvested and subjected to western blotting analysis with the indicated antibodies (*F*). *G*–*K*, NUDT16 suppresses SETD3 ubiquitination and protein degradation. HeLa cells were transfected with the indicated siRNAs for 24 h followed by transfection with SFB-SETD3 and HA-ubiquitin plasmids for another 24 h. Then, MG132 (10 μM) was added to the cells and incubated for 6 h, and the harvested cell lysates were subjected to Co-IP and western blotting assays with the indicated antibodies (*G*). The indicated plasmids were cotransfected into HeLa cells for 24 h followed by incubation with MG132 (10 μM) for another 6 h, and the cell lysates were subjected to Co-IP and western blotting analysis (*H*). The indicated constructs were cotransfected into HeLa cells for 24 h, followed by incubation with MG132 (10 μM) for another 6 h, and the cell lysates were subjected to Co-IP using anti-V5 tag beads and western blotting analysis (*I*). *J* and *K*, NUDT16 wild-type and knockout HeLa cells were incubated with 10 μg/ml cycloheximide (CHX) for the indicated periods of time. The cell lysates were harvested and analyzed by western blotting assay (n = 3) (*J*). Quantification of SETD3 protein levels from (*J*) using ImageJ software.

Growing evidence has demonstrated that PARylation can function as a priming signal for subsequent ubiquitination and facilitate the degradation of PARylated proteins (13, 26, 43). We sought to determine whether SETD3 protein stability is regulated by NUDT16-mediated PARylation, and a Co-IP assay was performed. As shown in Figure 5*A*, NUDT16 depletion significantly increased the level of PARylated SETD3. Consistent with these observations, ectopic expression of NUDT16 greatly reduced the levels of PARylated SETD3 (Fig. 5*B*). To explore whether NUDT16-mediated PARylation regulates the protein stability of SETD3, we generated NUDT16-knockout cells by CRISPR/Cas9 gene-editing technology. As shown in Figure 5*C*, the depletion of endogenous NUDT16 led to a dramatic decrease in the protein level of SETD3. Consistently, overexpression of NUDT16 markedly upregulated the protein level of SETD3 in a dose-dependent manner (Fig. 5*D*). Notably, the results of quantitative real-time PCR showed that the mRNA levels of SETD3 were not obviously altered after depletion of NUDT16 (Fig. S5*A*), further suggesting that NUDT16 modulates the protein stability of SETD3 at the posttranslational level.

Given that the protein level of NUDT16 exhibited the same expression patterns as SETD3, we wondered whether NUDT16 regulated the protein stability of SETD3 in a cell cycle-dependent manner and whether NUDT16-deficient cells were synchronized at each phase (Fig. S5, *B* and *C*). As shown in Figure 5, *E* and *F*, either knockdown or knockout of endogenous NUDT16 consistently resulted in a marked decrease in the SETD3 protein levels in each phase, indicating that NUDT16 could protect SETD3 from ubiquitination-mediated degradation in a cell cycle-independent manner.

To determine whether NUDT16 regulates the protein stability of SETD3 *via* the ubiquitin-proteasome pathway, *in vivo* ubiquitination assays were performed. Either depletion or knockdown of endogenous NUDT16 dramatically enhanced the polyubiquitination of SETD3, whereas ectopic expression of NUDT16 significantly reduced the ubiquitintion of SETD3 (Figs. 5, *G* and *H* and S5*D*). Importantly, overexpression of the catalytically inactive mutant of NUDT16 did not decrease the level of polyubiquitinated SETD3 (Fig. 5*I*), further confirming that NUDT16 hydrolase activity is required for inhibiting the ubiquitination of SETD3. In general, K11/K48 linkage-mediated ubiquitin chains label a target protein for degradation *via* the 26S proteasome, whereas K63-linked polyubiquitylation mainly affects the function of polyubiquitinated substrates (45). To investigate the type of Ub chain linkages that were added to SETD3 by NUDT16, an SFB-tagged SETD3 construct, together with a variety of HA-tagged ubiquitin mutants, was transfected into NUDT16-deficient 293T cells, and an *in vivo* ubiquitination assay was performed. As shown in Fig. S5*E*, NUDT16 depletion greatly increased K48-linked polyubiquitylation but did not alter any other chain-linked (K6, K11, K27, K29, K33, or K63) poly-Ub. Consistent with this notion, the depletion of NUDT16 resulted in a shortened half-life of endogenous SETD3 (Fig. 5, *J* and *K*). Collectively, these results demonstrate that NUDT16 promotes the dePARylation of SETD3 and enhances its protein stability by reducing the level of K48-linked polyubiquitylation.

### PARP1 interacts with SETD3 and promotes its PARylation and degradation

Poly(ADP-ribosyl)ation (PARylation), which is a key type of posttranslational modification, is mainly catalyzed by four members of the PARP family, namely, PARP1, PARP2, TNKS1 (PARP5a), and TNKS2 (PARP5b) (46). To determine which PARP contributes to the PARylation of SETD3, a Co-IP assay was performed. As shown in Fig. S6, *A*–*C*, an endogenous complex containing PARP1/SETD3 or TNKS1/2 and SETD3 was clearly detected. To further confirm whether PARP1 regulates the PARylation and protein stability of SETD3, we knocked out endogenous PARP1 by CRISPR/Cas9 gene-editing technology. Depletion of PARP1 markedly reduced the expression of PARylated SETD3 and greatly increased the total protein level of SETD3 (Fig. S6, *D* and *E*). Consistent with this observation, the half-life of the endogenous SETD3 protein was extensively increased in PARP1-knockout cells after treatment with cycloheximide (CHX) (Fig. S6, *F* and *G*).

It has been well documented that the ubiquitin‒proteasome pathway is responsible for the regulation of PARylated protein stability (13, 26, 27, 43). We sought to investigate whether PARP1 regulates SETD3 protein stability by regulating its ubiquitination, and an *in vivo* ubiquitination assay was performed. As shown in Fig. S6*H*, the knockdown of endogenous PARP1 dramatically decreased the level of ubiquitinated SETD3, indicating that PARP1-mediated PARylation of SETD3 is crucial for SETD3 ubiquitination and protein degradation. Given that both PARP1 and NUDT16 displayed greater affinity for PARylated SETD3, we asked whether there is competition between PARP1 and NUDT16 for binding to PARylated SETD3. As shown in Fig. S6, *I* and *J*, the depletion of NUDT16 did not disrupt the association of PARP1 with SETD3, however, the interaction between NUDT16 and SETD3 was dramatically compromised following the depletion of PARP1, indicating that PARP1-mediated PARylation of SETD3 is required for its binding to NUDT16. Taken together, these results firmly suggest that PARP1 covalently PARylates SETD3 and negatively regulates its stability *via* the ubiquitin-mediated proteasomal pathway.

### The E3 ubiquitin ligase CHFR binds to PARylated SETD3 and promotes its ubiquitination and degradation

**Figure 6:**
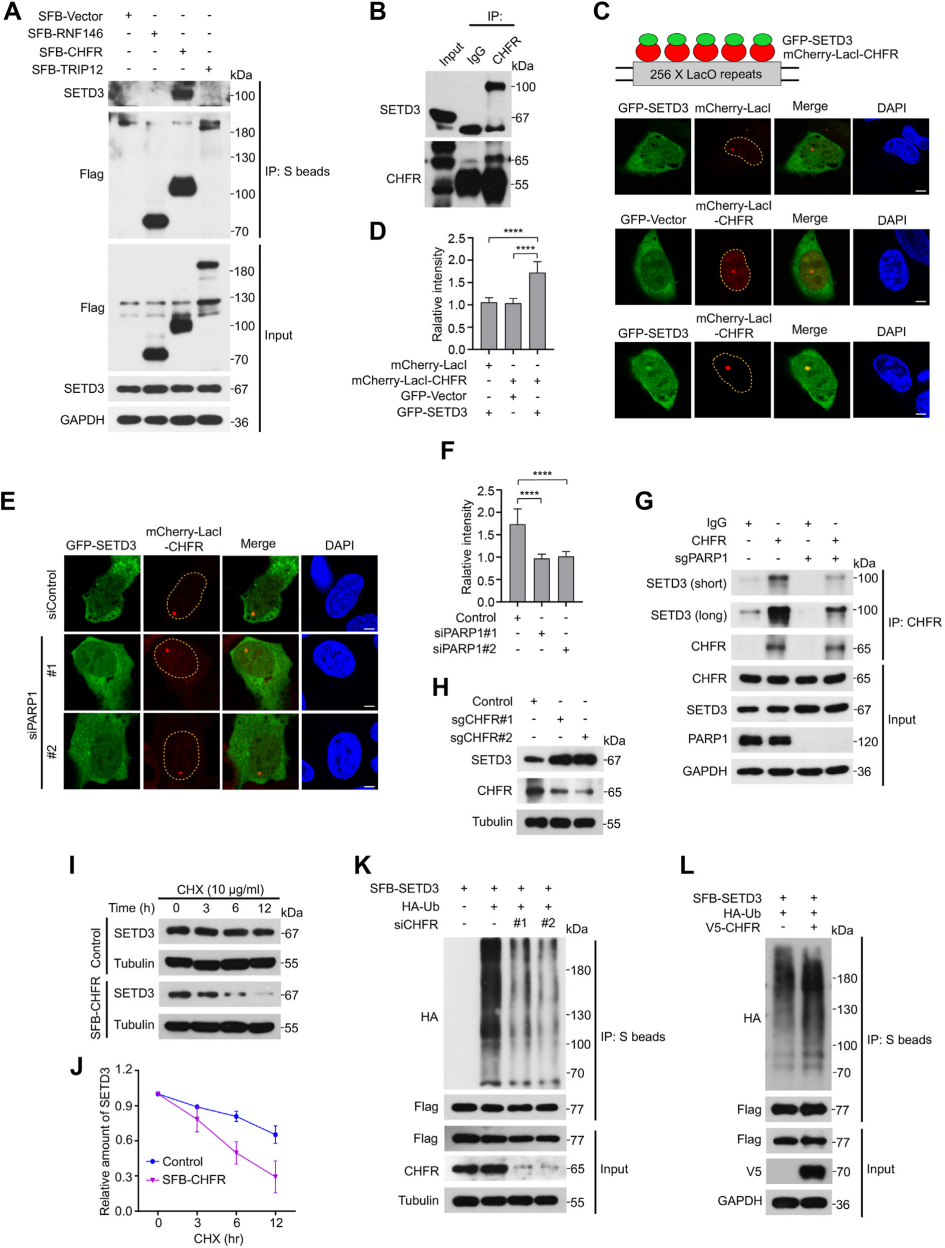
.**The E3 ubiquitin ligase CHFR binds to PARylated SETD3 and promotes its ubiquitination and degradation.***A* and *B*, the E3 ubiquitin ligase CHFR interacts with PARylated SETD3. HeLa cells were transiently transfected with the indicated plasmids for 24 h, and then, the cell lysates were subjected to Co-IP with anti-S beads followed by Western blotting analysis with the indicated antibodies (*A*). HeLa cells were lysed with NETN buffer and subjected to Co-IP using anti-IgG or anti-CHFR antibodies followed by western blotting analysis with the indicated antibodies (*B*). *C* and *D*, colocalization of GFP-SETD3 with mCherry-LacI-CHFR. U2OS cells with stable integration of LacO arrays (U2OS-265) were cotransfected with GFP-SETD3 and mCherry-LacI-CHFR for 24 h followed by immunostaining with DAPI and visualization by confocal microscopy (*C*). The fluorescence intensity of GFP-SETD3 foci was quantified using ImageJ software and normalized to the nuclear background (n > 30). The data are means ± SD of biological triplicate experiments. ∗∗∗∗*p* < 0.0001, Mann‒Whitney test. Scale bars, 5 μm (*D*). *E* and *G*, depletion of PARP1 impairs the interaction of SETD3 with CHFR. U2OS-265 cells were transfected with control siRNA or PARP1 siRNAs for 24 h followed by cotransfection of GFP-SETD3 and mCherry-LacI-CHFR for another 24 h, and the harvested cells were subjected to immunostaining and confocal microscopy imaging (*E*). The immunofluorescence intensity of GFP-SETD3 foci was quantified and normalized to the nuclear background (n > 30). The data are means ± SD of biological triplicate experiments. ∗∗∗∗*p* < 0.0001, Mann‒Whitney test. Scale bars, 5 μm (*F*). PARP1-depleted HeLa cells were subjected to a Co-IP assay using anti-IgG or anti-CHFR antibodies followed by western blotting analysis with the indicated antibodies (*G*). *H*–*L*, CHFR depletion suppresses SETD3 ubiquitination and enhances its protein stability. CHFR-knockout HeLa cells were lysed with NETN buffer and then subjected to western blotting with the indicated antibodies (*H*). HeLa cells were transfected with SFB-CHFR for 24 h followed by incubation with 10 μg/ml cycloheximide (CHX) for the indicated periods of time. The cell lysates were harvested and analyzed by western blotting assay (*I*). Quantification of SETD3 protein levels in (*J*) using ImageJ software. *K*, HeLa cells were transfected with the indicated siRNAs for 24 h followed by cotransfection of SFB-SETD3 and HA-ubiquitin plasmids for another 24 h, and then, MG132 (10 μM) was added and incubated for another 4 h. The harvested cell lysates were subjected to a Co-IP assay using anti-S beads and analyzed by Western blotting with the indicated antibodies. *L*, the indicated plasmids were cotransfected into HeLa cells for 24 h followed by incubation with MG132 (10 μM) for an additional 4 h. The cell lysates were subjected to a Co-IP assay using anti-S beads and analyzed by Western blotting with the indicated antibodies.

Accumulating evidence has revealed that PAR-binding motifs (PBMs), including macrodomains, PAR-binding zinc finger (PBZ) modules, and WWE domains, can bind to various forms of ADP-ribose moieties on PARP substrate proteins (47). Recent studies have shown that the E3 ligases CHFR (which contains PBZ modules), TRIP12, and RNF146 (which contains WWE domains) can interact with PARylated proteins and promote the degradation of these substrates *via* a ubiquitin‒proteasome-dependent mechanism (26, 48, 49). Therefore, we explored whether CHFR, TRIP12, or RNF146 also participate in the regulation of SETD3 protein stability. As shown in Figure 6*A*, a complex containing SETD3 and CHFR, but not TRIP12 and RNF146, was readily detected in 293T cells that were transfected with the indicated plasmids using co-IP experiments. Moreover, an endogenous co-IP assay was performed using anti-IgG or anti-CHFR antibodies. As shown in Figure 6*B*, an endogenous complex containing CHFR and SETD3 was clearly observed in HeLa cells. To further confirm these findings, we utilized the Lac operon-Lac repressor (LacO-LacI) anchoring system, as previously reported, to verify the interaction of SETD3 with CHFR (50). As shown in Figure 6, *C* and *D*, GFP-SETD3 efficiently colocalized with mCherry-LacI-CHFR in U2OS-265 cells carrying LacO arrays; however, GFP-SETD3 did not form visible foci in U2OS-265 cells expressing mCherry-LacI control plasmids, suggesting that SETD3 specifically interacts with genome-anchored CHFR but not with mCherry-LacI-mediated stalled replication forks.

Since the covalent addition of ADP-ribose units to target proteins is required for recognition by the E3 ligase CHFR, we sought to determine whether PARP1-mediated PARylation is essential for the recruitment of CHFR, and LacO-LacI reporter cells were employed again. Consistent with the above findings, depletion of endogenous PARP1 profoundly impaired the colocalization of GFP-SETD3 and mCherry-LacI-CHFR at LacO-anchored sites (Figs. 6, *E* and *F* and S7*A*). Moreover, knockout of PARP1 significantly reduced the interaction between CHFR and SETD3 according to a co-IP assay (Fig. 6*G*), indicating that PARP1-mediated PARylation was essential for the association of CHFR with SETD3.

To explore whether the E3 ligase CHFR regulates the protein stability of SETD3, half-life experiments and *in vivo* ubiquitination assays were performed. As shown in Figure 6*H*, CHFR depletion significantly increased endogenous SETD3 protein levels. Consistently, overexpression of CHFR significantly shortened the half-life of endogenous SETD3 relative to the control group (Fig. 6, *I* and *J*). Next, we investigated whether CHFR controls SETD3 protein stability through the ubiquitin‒proteasome pathway. As shown in Figure 6*K*, the knockdown of endogenous CHFR dramatically reduced the level of SETD3 polyubiquitination. In accordance with these observations, ectopic expression of CHFR greatly enhanced the extent of SETD3 polyubiquitination (Figs. 6*L* and S7*B*). Taken together, these results strongly suggest that the E3 ubiquitin ligase CHFR associates with PARylated SETD3 and promotes its ubiquitination and degradation.

### NUDT16 exerts a radioresistance effect by positively regulating SETD3 protein stability

**Figure 7:**
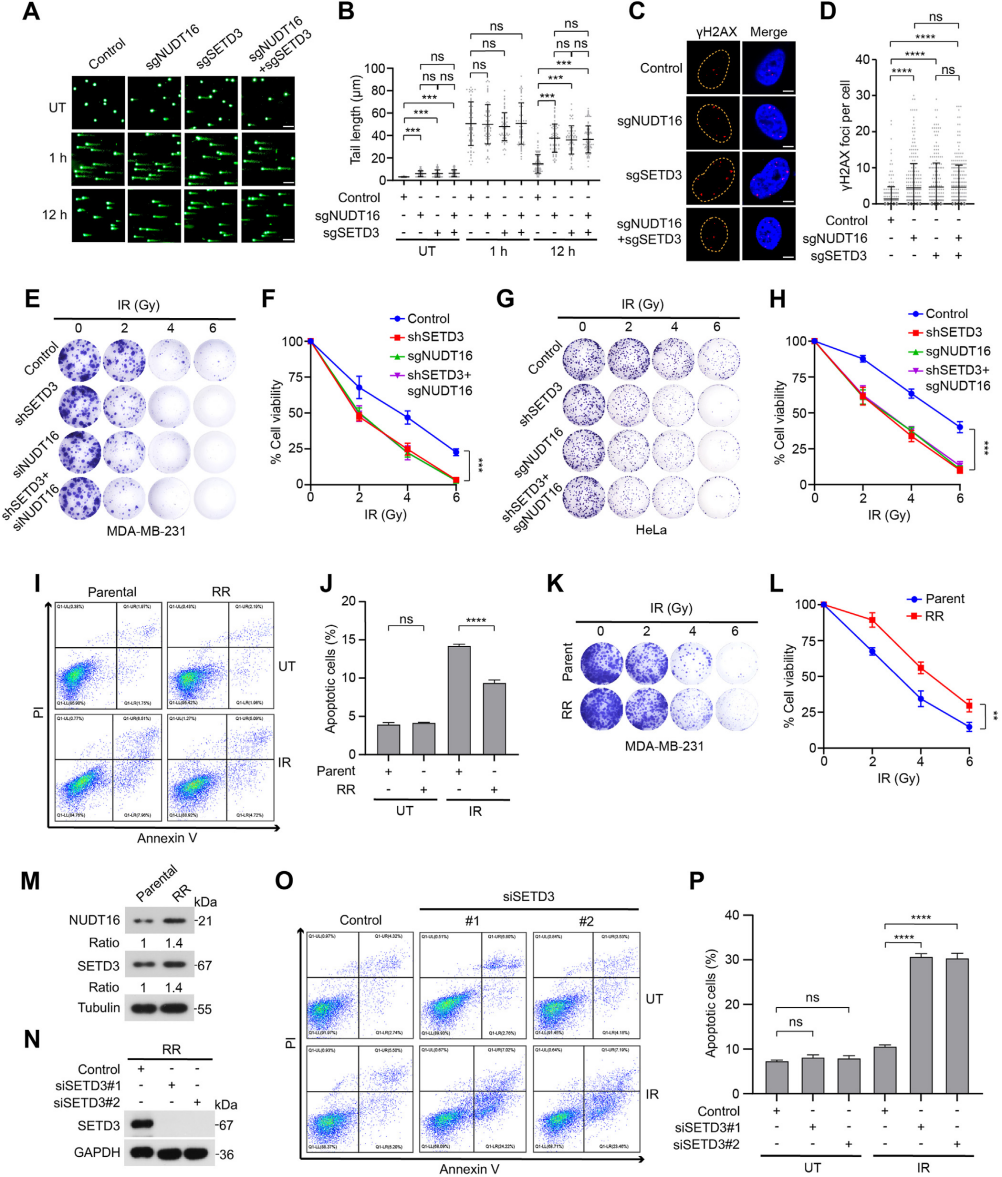
**NUDT16 exerts radioresistance effects *via* positive regulation of SETD3 expression.***A* and *B*, Control cells or indicated gene-deficient HeLa cells were either untreated or treated with IR (6 Gy) and allowed to recover for the indicated times followed by alkaline comet assay (*A*). Scale bar, 40 μm. The level of DNA breakage was determined by measuring the lengths of comet tail areas. The comet tail moment was quantified using CASP software. At least 60 comet tails were analyzed in each group (*B*). *C* and *D*, depletion of both NUDT16 and SETD3 did not further increase the accumulation of γH2AX foci. Representative micrographs showing the immunofluorescence assay results after the depletion of NUDT16, SETD3 or both in HeLa cells are shown in (*C*). Scale bar, 5 μm. Quantification of the γH2AX foci per cell using ImageJ software is presented in (*D*), and at least 100 cells were analyzed. *E*–*H*, depletion of both NUDT16 and SETD3 did not further enhance the sensitivity of either HeLa or MDA-MB-231 cells upon IR treatment. The indicated cells were first irradiated at different doses as indicated and subjected to a cell survival assay (*E* and *G*). The experiments were performed in triplicate, and the resulting colonies were quantified using ImageJ software. The results are shown as the averages of three independent experiments (*F* and *H*). *I*, parental or radiotherapy-resistant (RR) cells were either untreated or treated with IR (8 Gy) and then allowed to recover for 72 h, followed by Annexin V/PI staining and flow cytometry analysis. *J*, the results of apoptotic cell quantification in both groups are shown as the mean ± SD (n = 3). *K* and *L*, IR-resistant MDA-MB-231 cells exhibit stronger survival capability to IR treatment compared with the parental cells. The indicated cells were irradiated at different doses as indicated and then subjected to a cell survival assay (*K*). The resulting colonies were quantified using ImageJ software and the results are shown as the averages of three independent experiments (*L*). *M*, parental and RR cells were lysed with NETN buffer and analyzed by Western blotting with the indicated antibodies. *N*–*P*, the depletion of endogenous SETD3 in RR cells significantly increased the percentage of apoptotic cells after IR treatment. Control or SETD3-depleted RR cells were either untreated or treated with IR (8 Gy) and then allowed to recover for 72 h, followed by Annexin V/PI staining and flow cytometry analysis (*N* and *O*). *P*, results of apoptotic cell quantification in both groups are shown as the mean ± SD (n = 3).

Previous studies have shown that NUDT16 plays crucial roles in cell proliferation and DNA damage repair (13, 51). In this study, we found that SETD3 accelerated cell cycle S phase progression *via* replication stress resolution and promoted HR repair, resulting in cellular resistance to radiation therapy. Therefore, we sought to investigate whether NUDT16-mediated dePARylation of SETD3 contributes to the regulation of replication stress resolution and radioresistance in breast cancer cells, and a comet assay was performed. As shown in Figure 7, *A* and *B*, depletion of either NUDT16 or SETD3 led to a significant increase in the length of comet tails in both the untreated groups and the IR-treated groups. Importantly, depletion of both NUDT16 and SETD3 did not further enhance the tail length compared to depletion of either protein alone (Figs. 7, *A* and *B* and S8*A*), suggesting that SETD3 functions as a crucial downstream effector in mediating the role of NUDT16 in DNA damage repair. To determine the role of the NUDT16-SETD3 pathway in regulating replication stress, we examined the formation of endogenous γH2AX foci, and immunofluorescence assays were performed using anti-γH2AX antibodies. Accordingly, the numbers of γH2AX foci induced by the depletion of both NUDT16 and SETD3 were not further increased compared to those observed after the depletion of either NUDT16 or SETD3 alone (Fig. 7, *C* and *D*). Given that ATR kinase plays a critical role in the response to cellular replication stress and that targeting ATR has emerged as a promising strategy for the treatment of cancers that exhibit high levels of replication stress (52), we asked whether depletion of NUDT16 rendered cancer cells more sensitive to ATR inhibitors. As shown in Fig. S8, *C* and *D*, NUDT16-depleted cells were more cytotoxic than control cells and had attenuated colony formation ability after ATR inhibitor treatment, indicating that the NUDT16-SETD3 pathway might be an attractive target for synergizing with an ATR inhibitor to induce synthetic lethality through augmenting the levels of replication stress.

To determine the relationship between the biological functions of NUDT16 and SETD3, apoptosis and cell survival assays were performed. As shown in Figure 7, *E*–*H*, both NUDT16 depletion and SETD3 deletion greatly enhanced the sensitivity of HeLa and MDA-MB-231 cells to IR exposure. Importantly, depletion of both NUDT16 and SETD3 did not further affect the sensitivity of the cells to IR treatment compared with depletion of either NUDT16 or SETD3 alone (Figs. 7, *E*–*H* and S8*B*). Moreover, ectopic expression of NUDT16 greatly enhanced the resistance of cancer cells to IR treatment; however, depletion of SETD3 in these cells overexpressing NUDT16 largely reversed cell sensitivity towards IR exposure (Fig. S8, *E*–*G*). Together, these results indicated that SETD3 plays a critical role in mediating the functions of NUDT16 in cell proliferation and DNA repair processes.

To further elucidate whether the NUDT16-SETD3 pathway contributes to radiotherapy tolerance, we successfully established a radioresistant (RR) MDA-MB-231 cell line. As shown in Fig. S8*H*, the cell viability assay showed that the radioresistant cancer cells exhibited strong resistance to IR treatment. Moreover, a lower proportion of apoptotic cells was observed in IR-resistant cells than in parental cells after IR exposure (Fig. 7, *I* and *J*). A colony formation assay further confirmed that radioresistant MDA-MB-231 cells were more resistant to IR treatment than were the parental cells (Fig. 7, *K*–*L*). Notably, the protein levels of both NUDT16 and SETD3 were obviously upregulated in the IR-resistant cells (Fig. 7*M*). Therefore, we sought to determine whether depletion of SETD3 could reverse the sensitivity of these IR-resistant cells to irradiation, and a cell apoptosis assay was performed. As shown in Figure 7, *N*–*P*, the knockdown of endogenous SETD3 profoundly increased the ratio of apoptotic cells among the IR-resistant cells after IR treatment, reinforcing the notion that SETD3 is an important effector for the induction of radiotherapy tolerance.

## Discussion

Accumulating evidence has demonstrated that SETD3 performs oncogenic functions and plays crucial roles in regulating various biological events, including cell cycle progression, cell proliferation, and tumorigenesis (14, 17, 19, 37, 42). For instance, the USP27-mediated stability of the SETD3 protein promotes PLK1 gene transcription by binding to the promoter, resulting in cell proliferation and migration and HCC tumorigenesis (42, 53). It has also been reported that SETD3 interacts with and directly methylates the transcription factor FOXM1, leading to VEGF transcriptional silencing under normal conditions (16). Moreover, SETD3 specifically interacts with and facilitates p53 recruitment to its target gene in response to DNA damage, thereby rendering cancer cells more sensitive to doxorubicin treatment (14). However, the biological functions and underlying mechanisms by which SETD3 directly participates in the regulation of DNA replication stress and DNA damage repair and its roles in modulating sensitivity to irradiation in TNBC cells remain unclear.

In this study, we revealed that SETD3 regulates cell cycle progression and cell proliferation by decreasing R-loop-induced replication stress and enhances HR repair in response to DNA double-strand breaks, resulting in a substantial increase in the resistance of cancer cells to IR exposure (Figure 1, Figure 2, Figure 3). Moreover, we noted that the protein levels of endogenous SETD3 and the nudix hydrolase NUDT16 fluctuated throughout the cell cycle, exhibiting similar expression patterns and peaking in the S phase (Fig. 3). Mechanistically, we revealed that SETD3 associates with BRCA2 and promotes its recruitment to stalled replication fork and DNA damage sites upon replication stress or DNA double-strand breaks, respectively. Meanwhile, we demonstrated that NUDT16 binds to PARylated SETD3 and reverses SETD3 PARylation, thereby impairing the recognition and recruitment of the E3 ligase CHFR and enhancing SETD3 protein stability (Figs. 5 and 6). Importantly, depletion of endogenous SETD3 in NUDT16-deficient cells did not further exacerbate DNA breaks or enhance the sensitivity of cancer cells to IR treatment (Fig. 7). Notably, the protein levels of both NUDT16 and SETD3 were obviously upregulated in IR-resistant cells, and SETD3 depletion greatly increased the ratio of apoptotic cells among IR-resistant cells after IR treatment (Fig. 7), suggesting that the NUDT16-SETD3 pathway may play critical roles in the induction of radiotherapy tolerance.

Abnormal proliferation is the most typical characteristic of cancer cells, and increased replication stress inevitably occurs during DNA replication. To sustain chronic proliferation and survival, cancer cells have developed an evolutionarily conserved mechanism, namely, the replication stress response, which involves the activation of the ataxia telangiectasia mutated (ATM)- and Rad3-related (ATR)-mediated replication checkpoint, reshaping of stalled forks and employment of the DNA repair pathway (54). In general, replication stress originates from different sources, ranging from the formation of aberrant intrinsic structures of DNA templates (R-loops and G-quadruplex) to oncogene activation and transcription-replication conflicts (55). If cancer cells are unable to effectively respond to replication stress, they may bypass the G2/M checkpoint and undergo mitosis accompanied by overwhelming DNA errors, resulting in genomic instability as well as cell death induced by mitotic catastrophe (56). Our data showed that SETD3 could promote replication fork stability by recruiting BRCA2 to stalled replication forks and resolving R-loop structures independently of its methyltransferase activity, and that SETD3 could facilitate HR repair by interacting with BRCA2 and enhancing the recruitment of RAD51 proteins (Figs. 2 and 3), thereby accelerating cell cycle progression and rendering cells more sensitive to IR treatment (Fig. 1). It has been well documented that BRCA2 and members of the DEAD-box helicase (DDX) family play crucial roles in promoting DNA repair and replication fork stability *via* the regulation of R-loop accumulation (23, 57). We performed co-IP experiments using anti-SETD3 antibodies together with mass spectrometry analysis and identified BRCA2 and a variety of DDX family proteins, such as DDX17, DDX18, and DDX39, as potential factors that interact with SETD3 (Table S1 and Fig. S3*H*). Therefore, we speculated that DDX family members might also be involved in SETD3-mediated maintenance of the stability of stalled replication forks as well as promotion of homologous DNA repair, and future studies are needed to test this hypothesis.

PARylation, which is a crucial protein posttranslational modification, plays important roles in a variety of biological processes, including cell cycle progression, the replication stress response, and DNA damage repair, by modulating the protein stability of substrates or altering their subcellular localization and enzymatic activity (47). It has been demonstrated that four members of the PARP family, including PARP1, PARP2, TNKS1, and TNKS2, are involved in catalyzing the poly(ADP-ribosyl)ation of target substrates. Moreover, “erasers” of PARylation mainly include ten members of the dePARylation enzyme family, including PARG, MacroD1, MacroD2, TARG1, ARH1, ARH3, NUDT5, NUDT9, NUDT16 and ENPP1, and these proteins are responsible for removing the glycosidic bonds in PAR chains (58). In our study, we found that PARP1, TNKS1 and TNKS2 exhibited strong binding affinities for SETD3, and depletion of PARP1 greatly decreased the PARylation and stability of the SETD3 protein (Fig. S6). Given that the “writers” of PARylation PARP1 (nucleus localization preferred), TNKS1 and TNKS2 (cytoplasmic localization preferred) play distinct roles in regulating targeted substrates due to their different subcellular distributions (43), it is conceivable that the PARP1-mediated PARylation of SETD3 may contribute to its protein stability within nuclear compartments, whereas TNKS1 and TNKS2 may be responsible for catalyzing the PARylation of SETD3 in the cytoplasm (Fig. 6). Importantly, we also investigated the dePARylation enzymes that are involved in the hydrolyzation of PAR chains on PARylated SETD3 and found that NUDT16 exhibited stronger binding affinity for SETD3, while PARG, MacroD1 and MacroD2 showed moderate affinity for SETD3 (Fig. 4*C*). Notably, PARG is the major dePARylase that is responsible for hydrolyzing the glycosidic bonds between ADP-ribose moieties in PAR chains to eventually cause MARylation, and MacroD1 and MacroD2 are involved in the removal of MARylation at Asp/Glu-linkage bonds in target proteins (47). NUDT16 belongs to the Nudix superfamily of hydrolases and is primarily responsible for the digestion of phosphodiester bonds of PARylated or MARylated proteins, leading to the retention of ribose-5′-phosphate units on acceptor proteins (59). Therefore, NUDT16-mediated removal of phosphodiester bonds and PARG-mediated hydrolysis of O-glycosidic bonds may be responsible for the digestion of PARylated SETD3, and the resulting MARylated SETD3 might be attacked by MacroD1 and MacroD2, which completely removes the remaining mono-ADP-ribose units.

Numerous studies have reported that PARylation can also act as a signal to activate the ubiquitin–proteasome pathway and facilitate the degradation of ADP-ribosylated proteins. For instance, the E3 ligase RNF146 can recognize PARylated Axin and BRD7 *via* its WWE domain and target these proteins for degradation in a ubiquitin‒proteasome-dependent manner, resulting in increased activity of either the Wnt/β-catenin signaling pathway or the PI3K-Akt signaling pathway (26, 60). Moreover, the PAR-binding zinc finger (PBZ)-containing E3 ligase CHFR interacts with and degrades many PARylated substrates, including PLK1, RNF126, and PARP1, and plays crucial roles in cell cycle progression, DNA repair, and chemotherapy resistance (27, 61). Our study revealed that CHFR functions as a novel regulator of the recognition and degradation of PARylated SETD3, suggesting that the E3 ligase CHFR may also play an important role in mediating the development of radiotherapy tolerance in TNBC cells (Fig. 6). Consistent with the findings of previous reports (18), the protein levels of endogenous SETD3 fluctuated throughout the entire cell cycle, peaking in the S phase and gradually decreasing in the G2 and M phases (Fig. 4*A*). Notably, GSK3β-mediated phosphorylation of SETD3 is a prerequisite for FBXW7β-dependent ubiquitination and protein degradation of SETD3, thereby attenuating its oncogenic role in the proliferation of liver cancer cells (18). In the present study, we revealed that the dePARylase NUDT16-mediated dePARylation of SETD3 is important for impairing the interaction of the E3 ligase CHFR with SETD3, enhancing SETD3 protein stability and accelerating cell cycle progression (Figs. 5 and 6). Therefore, we speculated that this GSK3β-FBXW7β-dependent mechanism mainly plays its roles during the G2-M-G1 phase to promote the protein degradation of SETD3, whereas the NUDT16-CHFR-mediated pathway primarily enhances SETD3 protein stability during the S phase; thus, a rigorous and complicated mechanism is underpinned by the modulation of SETD3 protein levels throughout the entire cell cycle.

**Figure 8:**
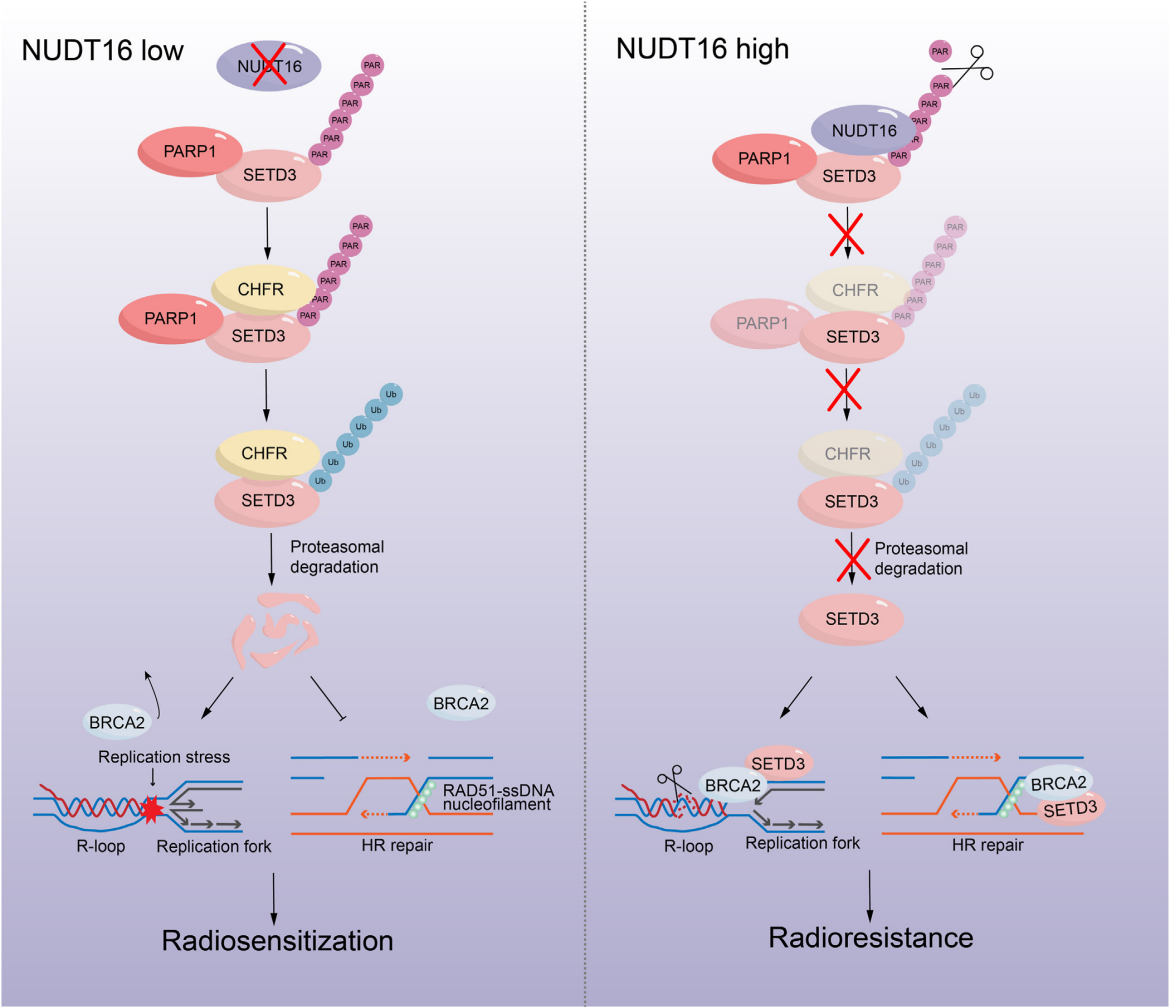
**Schematic model of the role of the NUDT16-SETD3 pathway in the regulation radiotherapy tolerance.** PARP1-mediated PARylation of SETD3 promotes the recruitment and interaction of the E3 ubiquitin ligase CHFR with SETD3, resulting in SETD3 degradation in a ubiquitin‒proteasome-dependent manner. Nevertheless, NUDT16-dependent dePARylation of SETD3 can block its ubiquitination and protein degradation and facilitate replication fork stability and HR repair by enhancing the recruitment of BRCA2 to stalled replication forks and DSB lesions, thereby significantly increasing cell proliferation and conferring resistance to radiotherapy.

In summary, our study demonstrated that SETD3 is an important regulator that promotes cell cycle progression by decreasing R-loop-induced replication stress and facilitating HR repair in a BRCA2-dependent manner. Depletion of SETD3 attenuates DNA replication progression and the stability of stalled replication forks and impairs HR repair, leading to increased radiotherapy sensitivity in TNBC cells. Moreover, we observed that the protein levels of SETD3 and the nudix hydrolase NUDT16 fluctuate throughout the entire cell cycle, and we revealed the potential mechanism by which NUDT16 recognizes PARylated SETD3 and reverses SETD3 PARylation, thereby impairing the recruitment of the E3 ligase CHFR for protein degradation and facilitating SETD3 protein stability. Importantly, depletion of endogenous SETD3 in NUDT16-deficient cells did not further exacerbate DNA breaks or enhance the sensitivity of cancer cells to IR treatment, suggesting that the NUDT16-SETD3 pathway might be a promising target for enhancing the sensitivity of cancer cells to radiotherapy in future clinical trials (Fig. 8).

## Data availability

All the data are available in the main text or the Supplementary materials. Mass spectrometry data has been deposited to the Proteome Xchange consortium under the dataset identifier PXD039367.

## Conflict of interest

The authors declare no potential conflicts of interest.
