# Supplementary material for: The dePARylase NUDT16 promotes radiation resistance of cancer cells by blocking SETD3 for degradation via reversing its ADP-ribosylation
Source: J Biol Chem. 2024 Jan 23;300(3):105671. doi: 10.1016/j.jbc.2024.105671 (PMC10926213; doi:10.1016/j.jbc.2024.105671)
Supplement: Supporting Figures S1–S8 [file mmc2.docx]

**Supplementary Information**

**The dePARylase NUDT16 promotes radiation resistance of cancer cells by blocking SETD3 for degradation via reversing its ADP-ribosylation**

Weijun Wu^1,2#^, Wenjing Wu^1,3#^, Yingshi Zhou^1,4#^, Qiao Yang^2#^, Shuting Zhuang^1^, Caixia Zhong^1^, Wenjia Li^5^, Aixin Li^2^, Wanzhen Zhao^1,2^, Xiaomin Yin^1,2^, Xuyu Zu^6^, Carmen Chak-Lui Wong^7^, Dong Yin^1^*, Kaishun Hu^1^*, Manbo Cai^2*^

^1^Guangdong Provincial Key Laboratory of Malignant Tumor Epigenetics and Gene Regulation, Guangdong-Hong Kong Joint Laboratory for RNA Medicine, Medical Research Center, Sun Yat-Sen Memorial Hospital, Sun Yat-Sen University, Guangzhou 510120, China

^2^Department of Oncology Radiotherapy, the First Affiliated Hospital, Hengyang Medical School, University of South China, Hengyang 421000, Hunan, China

^3^Department of Breast Oncology, Sun Yat-Sen Memorial Hospital, Sun Yat-Sen University, Guangzhou 510120, China

^4^Department of Ultrasound, Sun Yat-Sen Memorial Hospital, Sun Yat-Sen University, Guangzhou 510120, China

^5^Department of Pathology, The First Affiliated Hospital, Zhengzhou University,

Zhengzhou 450052, China

^6^Cancer Research Institute, The First Affiliated Hospital, Hengyang Medical School, University of South China, Hengyang, Hunan, 421001, China.

^7^Li Ka Shing Faculty of Medicine, Department of Pathology, The University of Hong Kong, Hong Kong, Guangdong, China

^#^These authors contributed equally to this work

*Corresponding author.

E-mail address: caimanbo@nhfyyy.com

E-mail address: [huksh3@mail.sysu.edu.cn](mailto:huksh3@mail.sysu.edu.cn)

E-mail address: Yind3@mail.sysu.edu.cn

**Supplemental Figures and Figure legends**

**
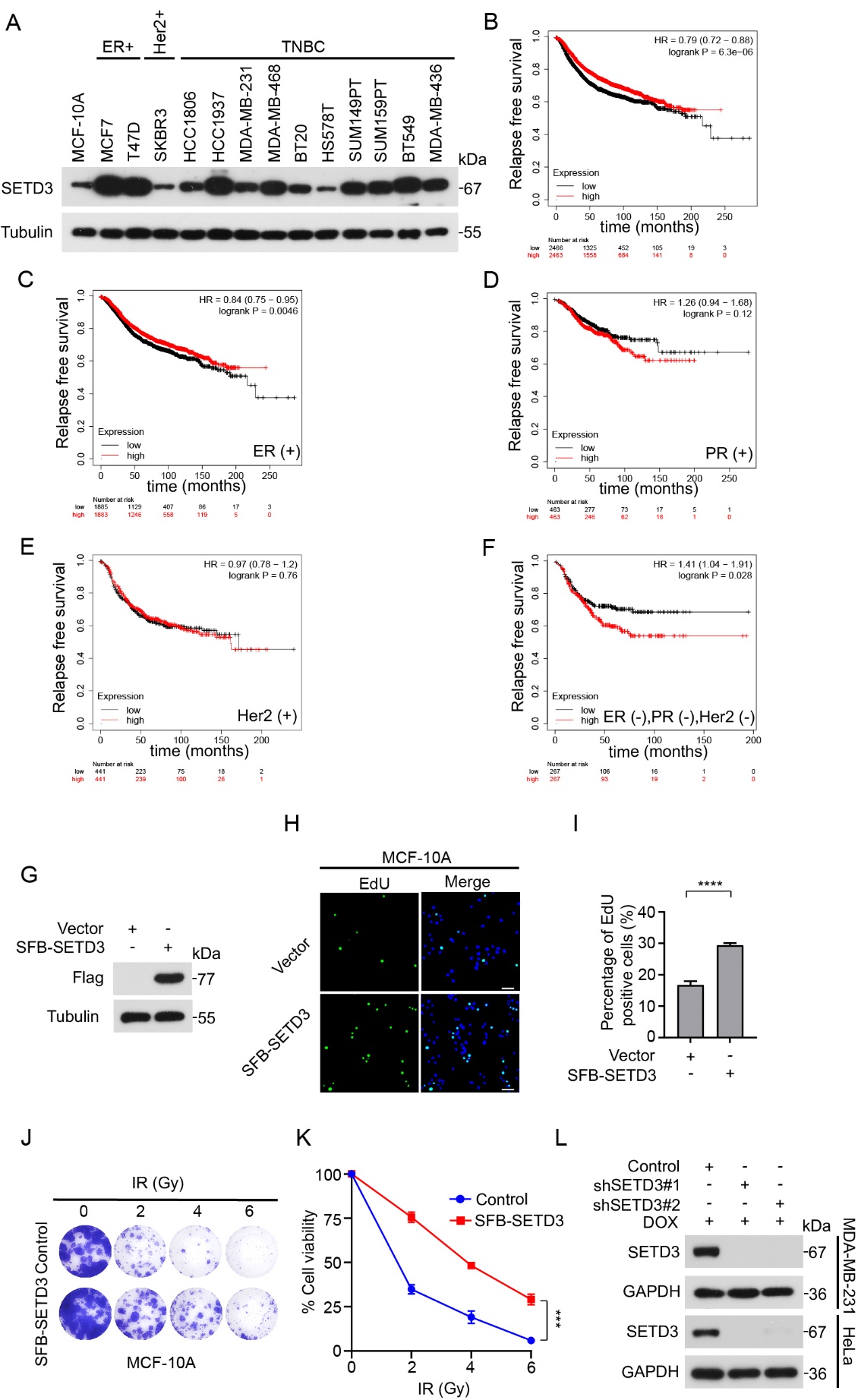
**

**Supplementary Figure 1**

**SETD3 promotes cell proliferation and radioresistance and is a prognostic marker for only TNBC patients.** (A) SETD3 protein levels in different cell lines were analyzed by western blot with indicated antibodies. (B-F) Survival analysis of the SETD3 protein levels in patients with different subtypes of breast cancer as classified by hormone receptor status. Kaplan‒Meier relapse-free survival curves were generated for (B) total breast cancer patients (n=4931), (C) estrogen receptor (ER)-positive patients, ER+ (n=3768), (D) progesterone receptor (PR)-positive patients, PR+ (n=926), (E) epidermal growth factor receptor 2 (Her2)-positive patients, Her2+ (n=882), and (F) ER, PR, and Her2 triple-negative patients (n=534). These analyses were performed based on data from the TCGA database. (G-I) SETD3 overexpression significantly facilitated S phase progression in MCF10A cells. SETD3 overexpressing cells (MCF10A) were incubated with EdU (10 μM) for 1 h followed by EdU-click assay with Alexa Fluor 488 azide staining (n = 3). Representative images of EdU-positive cells are shown in H. The percentage of EdU-positive cells was then quantified (I). (J-K) SETD3 expression promotes radiation resistance. MCF10A overexpressing SETD3 were irradiated with different dose of ionizing radiation, and cell survival was measured by colony-formation assays. Data represent the mean ± SD from three independent experiments. ***p < 0.001; **p < 0.01; *p < 0.05; ns, not significant. (L) HeLa and MDA-MB-231 cells stably expressing inducible SETD3 shRNA were treated with 1 μg/ml doxycycline (DOX) for 48 h and cell lysates were subjected to Western blot analysis.

**
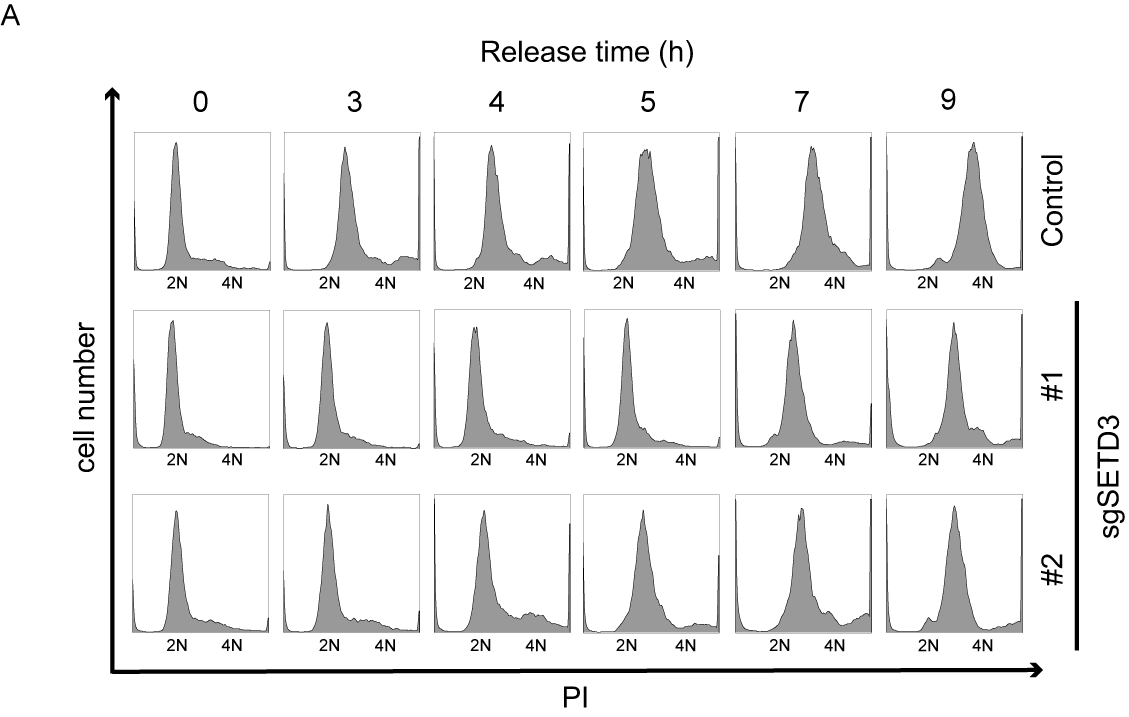
Supplementary Figure 2**

**Depletion of endogenous SETD3 delays S phase progression.** Wild-type and SETD3-deficient HeLa cells were synchronized at the G1/S phase boundary by HU (1 mM) treatment for 24 h and then released. The cells were harvested at the indicated time points and subjected to PI staining and flow cytometry analysis.


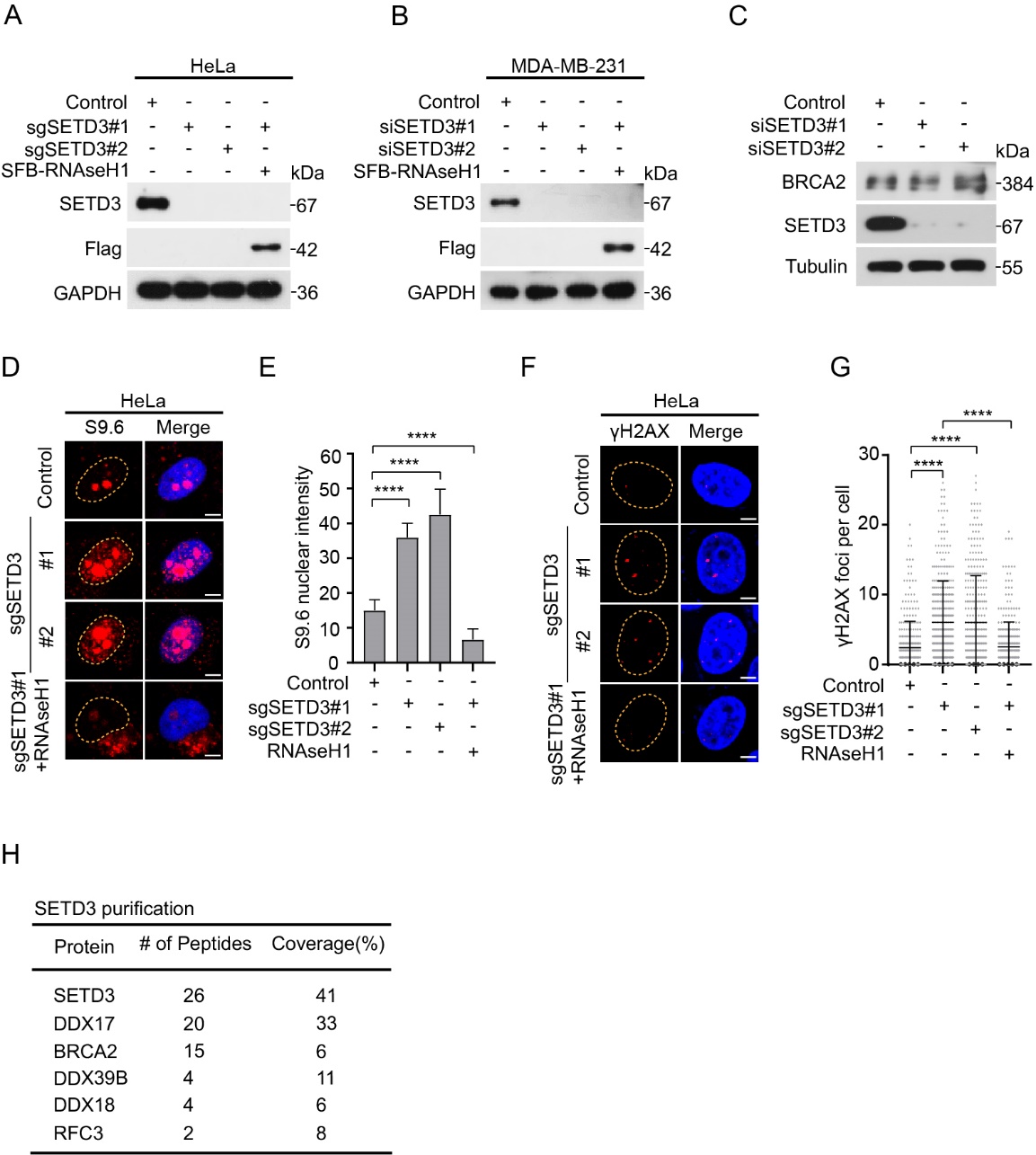


**Supplementary Figure 3**

(A) SETD3-deficient HeLa cells were transfected with either the empty vector or SFB-RNaseH1 for 24 h, and the cell lysates were subjected to western blotting analysis with the indicated antibodies. (B) MDA-MB-231 cells were first transfected with the indicated siRNA for 24 h and then transfected with the indicated plasmids for another 24 h. The harvested cells were lysed with RIPA buffer and subjected to western blotting analysis with the indicated antibodies. (C) HeLa cells were transfected with the indicated siRNAs for 48 h and then protein levels were examined by western blotting analysis with the indicated antibodies. (D-E) Depletion of SETD3 led to a substantial increase in nucleolar S9.6 signals. Both control and indicated HeLa cells were subjected to immunofluorescence assay using anti-S9.6 antibodies, and a representative image is shown in D. The nuclear S9.6 signal intensity was quantified by ImageJ software, and at least 100 cells were analyzed (E). (F-G) Depletion of SETD3 resulted in accumulation of γH2AX foci in HeLa cells. Representative micrographs showing the immunofluorescence assay results of SETD3-deficient cells are shown in F. Quantification of the γH2AX foci per cell using ImageJ software is shown in G, and at least 100 cells were analyzed. (H) Table summarizes proteins identified by mass spectrometry analysis.


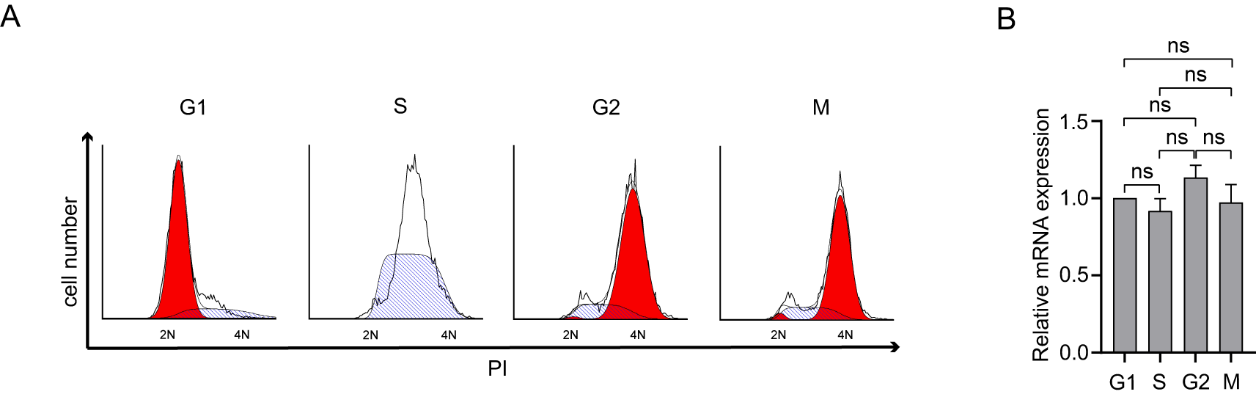


**Supplementary Figure 4**

(A) HeLa cells were synchronized at the G1/S phase boundary by HU (1 mM) treatment for 24 h and then released at the indicated times. The cells were harvested at the indicated time points and subjected to PI staining and flow cytometry analysis. (B) The harvested cells were subjected to qRT‒PCR with the indicated primers to measure the relative mRNA expression of SETD3.


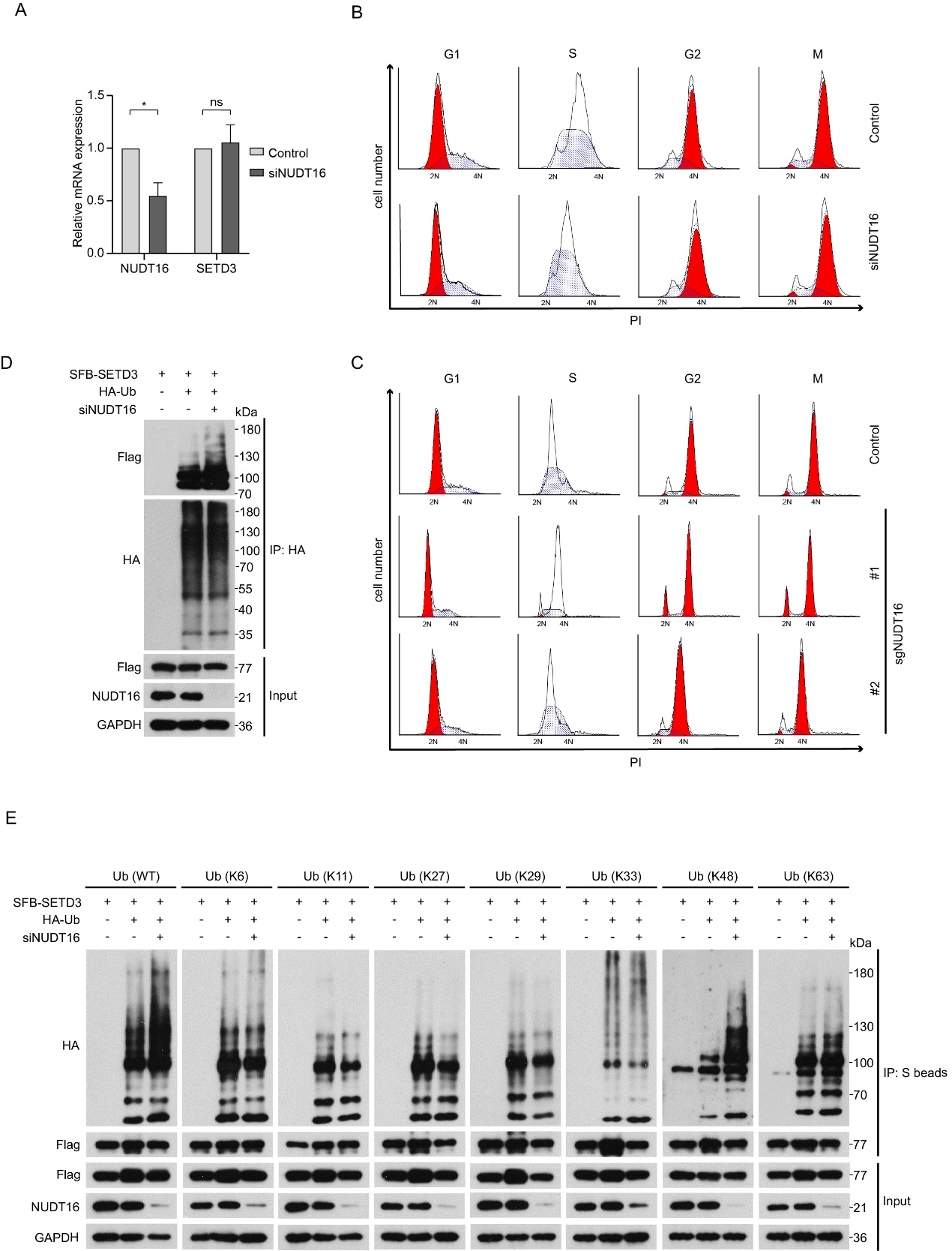


**Supplementary Figure 5**

(A) HeLa cells were transfected with NUDT16 siRNAs for 48 h and subjected to qRT‒PCR to monitor the relative mRNA expression of NUDT16 and SETD3. (B and C) Control and NUDT16-knockdown or NUDT16-knockout HeLa cells were synchronized at the G1/S boundary by HU (1 mM) treatment for 24 h and then released at the indicated times. The harvested cells were subjected to PI staining and flow cytometry analysis. (D) The depletion of NUDT16 increased SETD3 ubiquitination. HeLa cells were transfected with the indicated siRNAs for 24 h followed by transfection with SFB-SETD3 and HA-ubiquitin plasmids for another 24 h, and then, MG132 (10 μM) was added and incubated for an additional 4 h. The harvested cells were lysed with NETN buffer followed by Co-IP assay and western blotting analysis with the indicated antibodies. (E) NUDT16 depletion greatly increased the K48-linked poly-Ub levels of SETD3. 293T cells were transfected with the indicated siRNAs for 24 h followed by transfection of the indicated plasmid for another 24 h, and then, MG132 (10 μM) was added and incubated for an additional 4 h. The cells were lysed with NETN buffer and subjected to Co-IP as well as western blotting analysis with the indicated antibodies.


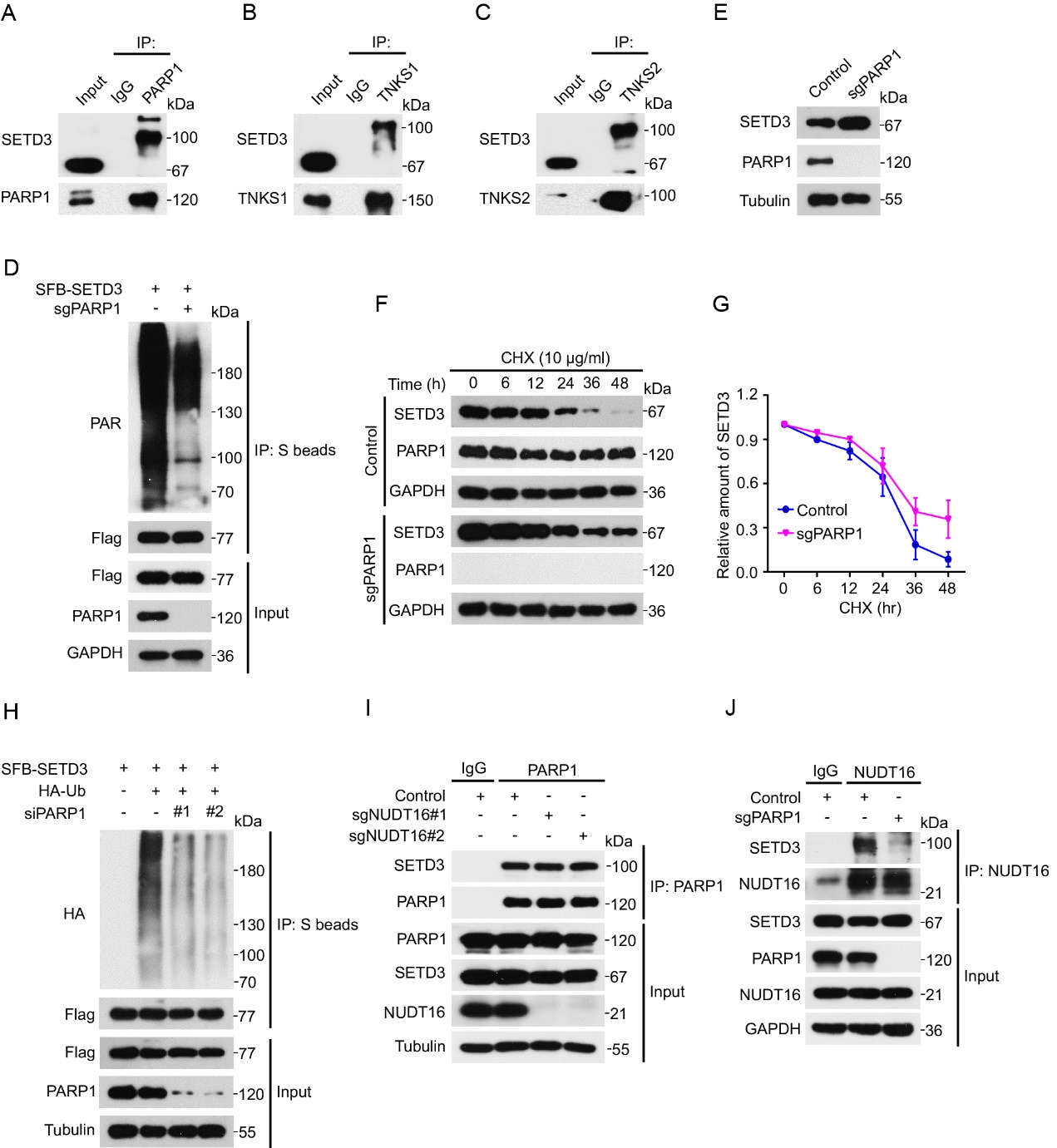


**Supplementary Figure 6**

(A-C) SETD3 interacts with PARP1, TNKS1 and TNKS2. HeLa cell lysates were subjected to co-IP assay followed by western blotting analysis with the indicated antibodies. (D) PARP1 knockout significantly reduced the PARylation of SETD3. PARP1-depleted HeLa cells were transiently transfected with SFB-SETD3 for 24 h and lysed with NETN buffer, followed by Co-IP using anti-S beads and western blotting analysis with the indicated antibodies. (E) PARP1 depletion enhances SETD3 protein stability. PARP1 knockout HeLa cells were lysed with NETN buffer, followed by western blotting analysis with the indicated antibodies. (F and G) Depletion of PARP1 extended the half-life of endogenous SETD3. WT and PARP1-KO HeLa cells were incubated with 10 μg/ml cycloheximide (CHX) for the indicated periods of time. Cell lysates were harvested and analyzed by Western blotting with the indicated antibodies (n=3) (F). Quantification of SETD3 protein levels from (F) using ImageJ software. The error bars indicate SEM. Relative expression levels were normalized to the SETD3 protein expression level at 0 h (G). (H) PARP1 depletion dramatically reduced the polyubiquitination of SETD3. HeLa cells were transfected with the indicated siRNAs for 24 h, followed by transfection of SFB-SETD3 and HA-ubiquitin plasmids for another 24 h; MG132 (10 μM) was added and incubated for 4 h, and the harvested cells were lysed with NETN buffer, followed by Co-IP and western blotting analysis with the indicated antibodies. (I) Depletion of NUDT16 did not affect the binding of SETD3 with PARP1. Wild-type and NUDT16-knockout HeLa cells were lysed with NETN buffer, followed by Co-IP and western blotting analysis with the indicated antibodies. (J) Depletion of PARP1 significantly inhibited the association between SETD3 and NUDT16. Wild-type and PARP1-knockout HeLa cells were lysed with NETN buffer, followed by Co-IP using either anti-IgG or anti-NUDT16 antibodies and western blotting analysis with the indicated antibodies.


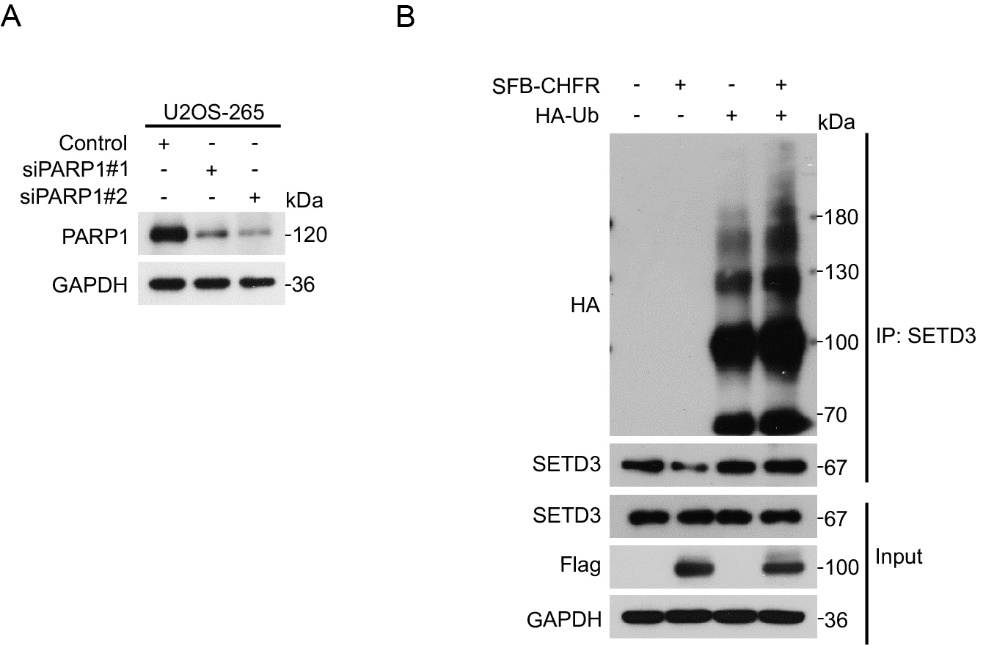


**Supplementary Figure 7**

(A) U2OS-265 cells were transfected with the indicated PARP1 siRNAs for 48 h, and protein levels were examined by western blotting analysis with the indicated antibodies. (B) Overexpression of CHFR promotes SETD3 ubiquitination. The indicated plasmids were cotransfected into HeLa cells for 24 h, and then, MG132 (10 μM) was added and incubated for an additional 4 h, followed by a co-IP assay using anti-SETD3 antibodies and western blotting analysis with the indicated antibodies.


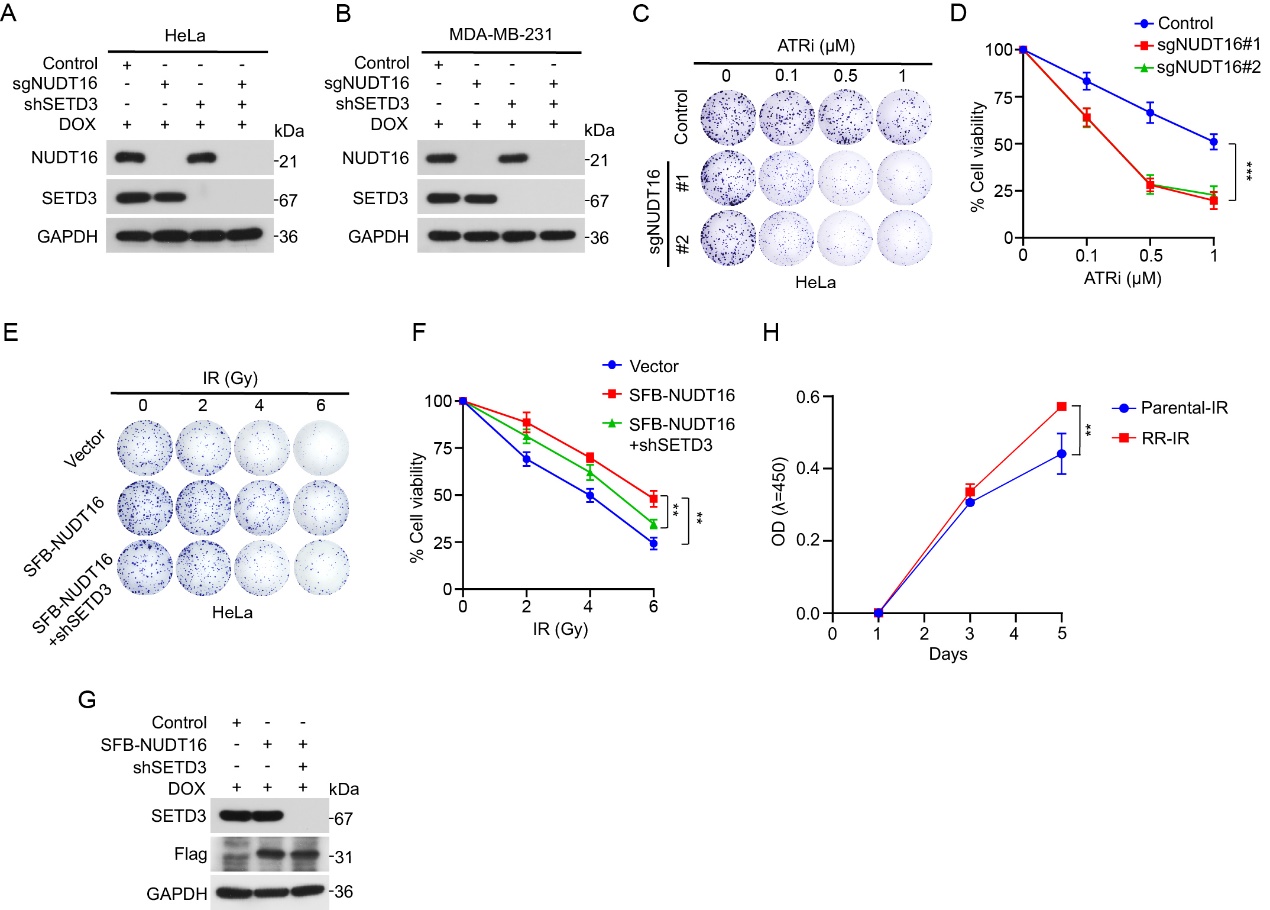


**Supplementary Figure 8**

(A) The indicated sgRNAs or shRNAs were transfected into HeLa cells, and the cell lysates were analyzed by western blotting assay with the indicated antibodies. (B) MDA-MB-231 cells were transfected with the indicated siRNAs or shRNAs for 48 h, and protein levels were examined by western blotting analysis with the indicated antibodies. (C-D) Depletion of NUDT16 enhances the sensitivity of HeLa cells to ATR inhibitor (VE-821) treatment. The indicated cells were incubated with VE-821 at different concentration as indicated for 24 h and subjected to a cell survival assay (C). The experiments were performed in triplicate, and the resulting colonies were quantified using ImageJ software. The results are shown as the averages of three independent experiments (D). (E-G) Depletion of SETD3 in HeLa cells overexpressing NUDT16 largely reversed cell sensitivity towards IR exposure. The indicated cells were irradiated at different doses as indicated and subjected to a cell survival assay (E). The experiments were performed in triplicate, and the resulting colonies were quantified using ImageJ software and the results are shown as the averages of three independent experiments (F). (G) HeLa cells stably integrated with SFB-NUDT16 plasmids were further transfected with the inducible SETD3 shRNA followed by treatment with 1 μg/ml doxycycline (DOX) for additional 48 h, then the cells were harvested and lysed with NETN buffer followed by western blotting analysis with the indicated antibodies. (H) CCK-8 assays were performed to observe the radiosensitivity of parental and radiotherapy-resistant (RR) cells after IR treatment (6 Gy). The data are presented as the mean ± SD of three independent experiments. **p<0.01.
